# Modulating gentamicin release from Poly(lactic acid) composite matrices by tuning shell-derived calcium carbonate content for local antibacterial delivery

**DOI:** 10.3389/fbioe.2026.1976612

**Published:** 2026-09-30

**Authors:** Anastassiya Khrustaleva, Dmitriy Khrustalev, Azamat Yedrissov, Irina Losseva, Ivan Chernykh, Artyom Savelyev, Marlen Kiikbayev, Vladimir Kazantsev

**Affiliations:** 1 Institute of Life Sciences, Karaganda Medical University, Karaganda, Kazakhstan; 2 School of Pharmacy, Karaganda Medical University, Karaganda, Kazakhstan; 3 Department of Pharmacology, Ryazan State Medical University, Ryazan, Russia

**Keywords:** biogenic calcium carbonate, gentamicin release, Higuchi model, local antibiotic delivery, mollusk shell powder, poly(lactic acid), transport kinetics

## Abstract

Controlling the release of hydrophilic antibiotics from biodegradable polyester matrices remains a key challenge in local antibacterial delivery. Here, we investigated whether gentamicin sulfate release from compact hot-pressed poly (lactic acid) (PLA) matrices can be modulated by varying the content of a shell-derived biogenic CaCO_3_-containing mineral phase. PLA composites containing a constant gentamicin sulfate loading of 5 wt% and 0, 10, 20, 30, or 40 wt% mineral phase were prepared and evaluated for gentamicin release in phosphate-buffered saline at pH 7.4 and 37 °C for 16 days. Release behavior was analyzed using mass and geometric normalization, multiple linear regression accounting for specimen thickness, and kinetic modeling of the early-stage and complete release profiles. Complementary FTIR, SEM/EDS, elemental, and water-uptake analyses were used to characterize the mineral phase, composite microstructure, and matrix interaction with the aqueous medium. Antibacterial activity of the hot-pressed matrices was additionally evaluated against *Staphylococcus aureus* and *Escherichia coli*. All compositions exhibited biphasic release, characterized by an initial rapid stage followed by slower release approaching a plateau. Increasing mineral-phase content was accompanied by a progressive increase in area-normalized gentamicin release (*Q*_*a*_ *)* and in the apparent Higuchi release constant (*K*_*H*_), which increased from 0.650 ± 0.044 to 2.007 ± 0.195 µg·mm^-2^·h^-1/2^ between the 0 and 40 wt% formulations. The relationship between *K*_*H*_ and mineral-phase content was strongly positive (*R* ^*2*^ = 0.9185, *p* < 0.0001), while the complete 0–16-day profiles were best described by an asymptotic first-order model. Importantly, mineral-phase content remained significantly and positively associated with mass-normalized gentamicin release after adjustment for specimen thickness, indicating that the composition-dependent effect could not be explained solely by geometric variability among the matrices. All investigated formulations retained pronounced antibacterial activity against both test microorganisms after hot pressing. These findings demonstrate that shell-derived mineral-phase content provides an effective compositional parameter for tuning gentamicin transport in compact PLA matrices without increasing antibiotic loading or chemically modifying the polymer. The combined release, kinetic, microstructural, and water-uptake data support a role of polymer–mineral organization and enhanced matrix–medium interaction in the observed composition-dependent release behavior while antibacterial functionality is retained after thermal processing.

## Graphical Abstract

**Figure:**
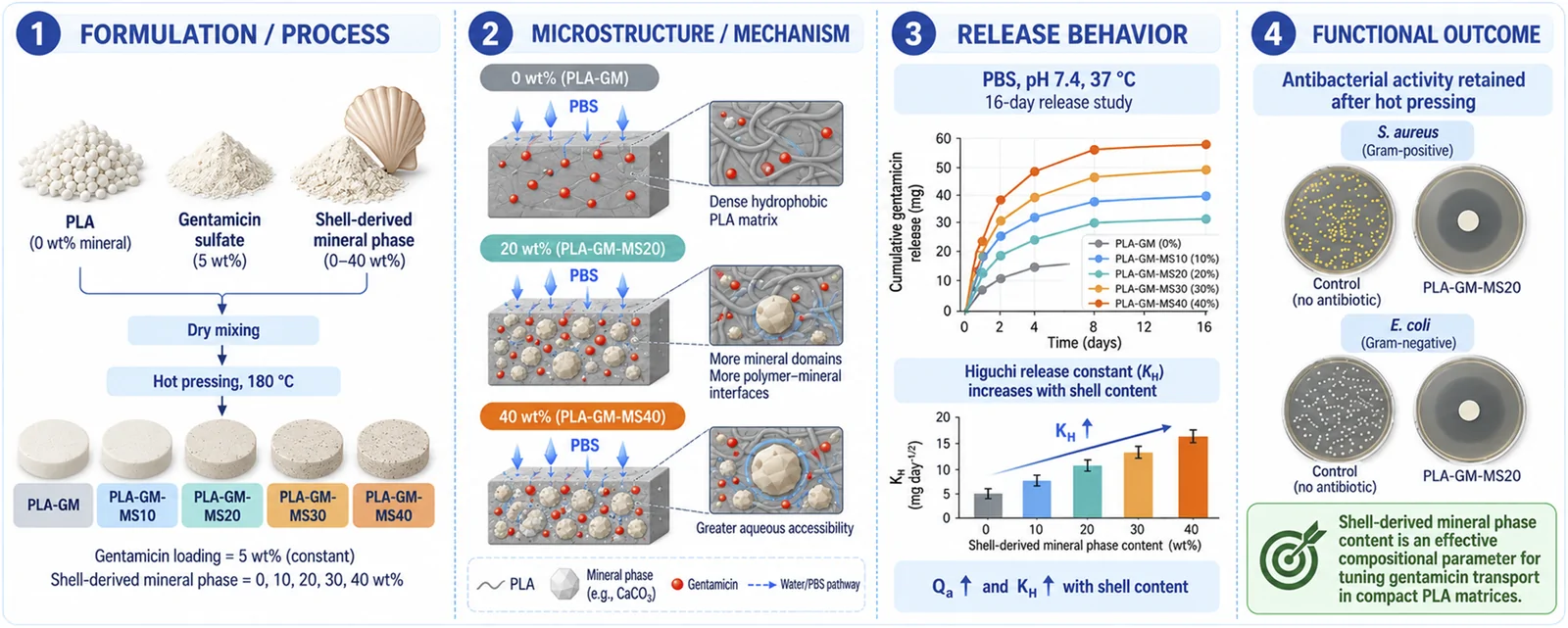

## 1 Introduction

Implantable materials have substantially expanded the capabilities of modern orthopedics, trauma surgery, and reconstructive surgery. However, their widespread use is still accompanied by one of the most challenging clinical problems: the development of implant-associated infections (Lu et al., 2022; Ter Boo et al., 2015). Unlike conventional wound infections, infections on implant surfaces develop under conditions in which microorganisms interact closely with a foreign material. In this environment, bacteria can rapidly adhere to the surface, form mature biofilms, and acquire increased tolerance to antibacterial therapy (Rozis et al., 2021; Steadman et al., 2023). As a result, even rational systemic antibiotic administration does not always provide therapeutically effective drug concentrations directly at the implantation site, particularly during the early postoperative period, when the risk of microbial colonization is highest (ter Boo et al., 2015). Therefore, the development of local antibiotic delivery systems is regarded as one of the most promising strategies for the prevention and treatment of implant-associated infections (ter Boo et al., 2015; Steadman et al., 2023; Wang et al., 2023).

Among the various platforms for local drug delivery, biodegradable polymeric materials occupy a special position because they can simultaneously provide structural support and serve as carriers for biologically active compounds (Chang et al., 2006; Pisani et al., 2019). Their use makes it possible to achieve high local antibiotic concentrations directly at the implantation site, reduce systemic drug exposure, and eliminate the need for carrier removal after completion of the therapeutic effect (Chang et al., 2006). Poly (lactic acid) (PLA) is one of the most extensively studied biodegradable polymers and is widely used in the development of temporary implants, tissue-engineering constructs, and controlled drug delivery systems because of its biocompatibility, biodegradability, adequate mechanical properties, and processability (Barcena et al., 2024). An additional advantage of PLA is its compatibility with extrusion, hot pressing, and additive manufacturing, which provides considerable technological flexibility in the design of medical devices (Barcena et al., 2024; Khrustaleva et al., 2026a). This is consistent with a recent systematic review of bioactive polymer composites for 3D-printed bone implants, in which PLA was identified as one of the key synthetic biodegradable matrices for bone-oriented materials (Khrustaleva et al., 2026b). At the same time, that review emphasized that most studies have focused primarily on osteoconductive mineral fillers and the mechanical and biological performance of composites, whereas antibacterial and transport-regulating functions remain less extensively investigated.

Therefore, despite the advantages of PLA as a processable and biocompatible matrix, its use as a carrier for hydrophilic drugs requires special consideration. The relatively hydrophobic nature of the polyester matrix restricts penetration of the aqueous medium into the bulk of the material. Consequently, the transport of hydrophilic compounds is governed not only by their physicochemical properties, but also by the internal organization of the material, including porosity, drug distribution, and the presence of water-accessible pathways (Chang et al., 2006; Yoo and Won, 2020). PLA-, PLGA-, and PCL-based systems often exhibit a multistage release profile, including a pronounced initial burst followed by a slower stage limited by dissolution, diffusion, swelling, or degradation of the polymer matrix (Chang et al., 2006; Yoo and Won, 2020). For gentamicin sulfate, a hydrophilic aminoglycoside antibiotic, this issue is particularly important because the efficacy of local antibacterial therapy is determined not only by the amount of drug incorporated into the matrix, but also by the fraction that becomes available for controlled release (Chang et al., 2006; Pisani et al., 2019).

A substantial body of literature on biodegradable systems for local antibiotic delivery has focused on the development of new polymeric matrices, chemical modification of materials, or the creation of porous architectures that enable modulation of drug release profiles (Chang et al., 2006; Yoo and Won, 2020; Fosca et al., 2021). Considerably less attention has been paid to the possibility of regulating antibiotic transport by modifying the internal organization of an existing polymer matrix without changing its chemical structure. In particular, it remains insufficiently understood whether a biogenic calcium-containing mineral phase can influence the transport properties of PLA composites in addition to its conventional role as a bioactive filler. From this perspective, varying the content of a biogenic mineral phase may be considered a potential technological parameter for controlling the transport of hydrophilic drugs without altering the chemical nature of the polymer matrix.

To test this concept, natural calcium-containing materials are of particular interest because they may influence the internal organization and transport behavior of polymer composites. One such material is mollusk shell powder, which consists predominantly of calcium carbonate present, depending on the biological source, as aragonite and/or calcite (Azarian and Sutapun, 2022; Gigante et al., 2020; Schiopu et al., 2025). In addition to its availability, environmental attractiveness, and potential bioactivity, mollusk shell powder is regarded as a promising component of bone-oriented biocomposites and polymeric materials containing natural mineral fillers (Azarian and Sutapun, 2022; Gigante et al., 2020). Shell-derived mineral fillers have also recently been incorporated into gentamicin-containing PLA systems for additive manufacturing applications (Khrustaleva et al., 2026d). Unlike synthetic calcium phosphate materials, such as hydroxyapatite, mollusk shells represent a naturally formed CaCO_3_-containing mineral system that may include small amounts of accompanying inorganic components, including Mg, Sr, Zn, Fe, and other trace elements. This compositional complexity makes shell powder of interest not only as a calcium source, but also as a natural mineral component that may potentially influence the bioactivity, interfacial organization, and transport properties of polymer composites (Hou et al., 2026; Chung et al., 2024).

However, its possible effect on the transport properties of biodegradable polymer systems remains insufficiently studied. In most previous studies, biogenic calcium carbonate or other calcium-containing mineral phases have been used mainly to enhance the bioactivity, osteoconductivity, or mechanical properties of materials (Gigante et al., 2020; Kanak et al., 2023). In contrast, in the present study, mollusk shell powder is considered primarily as a potential structural modifier of mass transport processes capable of altering the release kinetics of a hydrophilic antibiotic from a PLA matrix.

On this basis, we hypothesized that the content of a shell-derived biogenic CaCO_3_-containing phase may serve as a factor regulating mass transport in a compact PLA matrix. The aim of this study was to evaluate the effect of mollusk shell powder on the release kinetics of gentamicin sulfate from hot-pressed PLA composites and to quantitatively characterize their transport properties. For this purpose, composites with different mineral phase contents were prepared, antibiotic release profiles were investigated, and the transport characteristics were comparatively analyzed using area-normalized release and Higuchi kinetic modeling. This approach was designed to determine whether mineral-phase content can serve as a compositional parameter for modulating hydrophilic drug transport without changing the chemical nature of PLA or increasing drug loading.

## 2 Materials and methods

### 2.1 Materials

Poly (lactic acid) (PLA, NatureWorks Ingeo 4032D, USA) was used as the polymer matrix. Gentamicin sulfate was used as the antibacterial component. Phosphate-buffered saline (PBS, pH 7.4) was used as the release medium. Dimethyl sulfoxide (DMSO), ascorbic acid, and phosphate-buffered saline were purchased from Sigma-Aldrich (St. Louis, MO, USA).

Ground mollusk shells were used as the mineral filler. The mollusk shell material used in this study was supplied by ASKOR AgroTrade LLC (Buynaksk, Russia). The raw shell material was first cleaned to remove surface contaminants, thoroughly washed several times with distilled water, and dried in an oven at 100 °C. The dried shells were then mechanically milled to obtain a fine powder, which was subsequently used for the preparation of composite matrices.

### 2.2 Preparation and characterization of the mollusk shell powder

The mollusk shell material was mechanically milled and sieved through a 500 µm sieve, and the fraction passing through the sieve was used for composite preparation. The shell-derived powder was characterized by FTIR spectroscopy and scanning electron microscopy according to the procedures described in Sections 2.4 and 2.5, respectively. Representative particle dimensions were determined directly from the SEM micrographs.

The elemental composition of the shell-derived mineral powder was determined by inductively coupled plasma atomic emission spectroscopy (ICP-AES). For uniform presentation and comparison, the concentrations of the detected elements were expressed as wt%.

### 2.3 Preparation of composite matrices

Pre-milled PLA was mixed with mollusk shell powder and gentamicin sulfate according to the target composite formulations. The components were dry-mixed at room temperature for 5 min until a visually homogeneous mixture was obtained.

To evaluate the effect of mineral filler content on gentamicin release kinetics, a series of composite matrices containing 0, 10, 20, 30, and 40 wt% mollusk shell powder was prepared at a constant gentamicin sulfate loading of 5 wt%. The compositions of the investigated composite matrices are shown in Table 1.

**TABLE 1:** Composition of the composite matrices.

Sample	PLA, wt%	Mollusk shell powder, wt%	Gentamicin sulfate, wt%
PLA-GM	95	0	5
PLA-GM-MS10	85	10	5
PLA-GM-MS20	75	20	5
PLA-GM-MS30	65	30	5
PLA-GM-MS40	55	40	5

For consistency, the composite series is designated as PLA-GM-MS, where PLA, denotes poly (lactic acid), GM, denotes gentamicin sulfate, and MS, denotes mollusk shell powder. The formulation without mollusk shell powder is designated PLA-GM, while shell-containing formulations are designated PLA-GM-MS10, PLA-GM-MS20, PLA-GM-MS30, and PLA-GM-MS40, according to the shell powder content (wt%).

The resulting composite mixtures were hot-pressed using a Schulze GmbH Blue Press X Pneu laboratory heat press (Berlin, Germany) at 180 °C to obtain film specimens. The hot-pressing procedure was performed using a press with a working surface of 400 × 500 mm. The material was hot-pressed at 0.25 MPa for 30 s per cycle. To ensure complete consolidation while limiting prolonged thermal exposure of gentamicin, the pressing cycle was repeated up to three times, with brief cooling intervals between successive cycles. After pressing, the samples were cooled at room temperature. The thickness of the obtained films ranged from 0.35 to 0.85 mm and was measured using a micrometer. For each formulation, three separate specimens were fabricated in independent hot-pressing runs. Each specimen was individually formed and pressed and was not obtained by cutting replicate test pieces from a single common hot-pressed plate. Thus, the three specimens within each formulation represented independent fabrication replicates.

Each sample was placed in a separate tube so that it was completely immersed in the buffer solution while maintaining access of the main film surfaces to the medium throughout the incubation period. A schematic overview of PLA-GM-MS plate preparation is shown in Figure 1.

**FIGURE 1:**
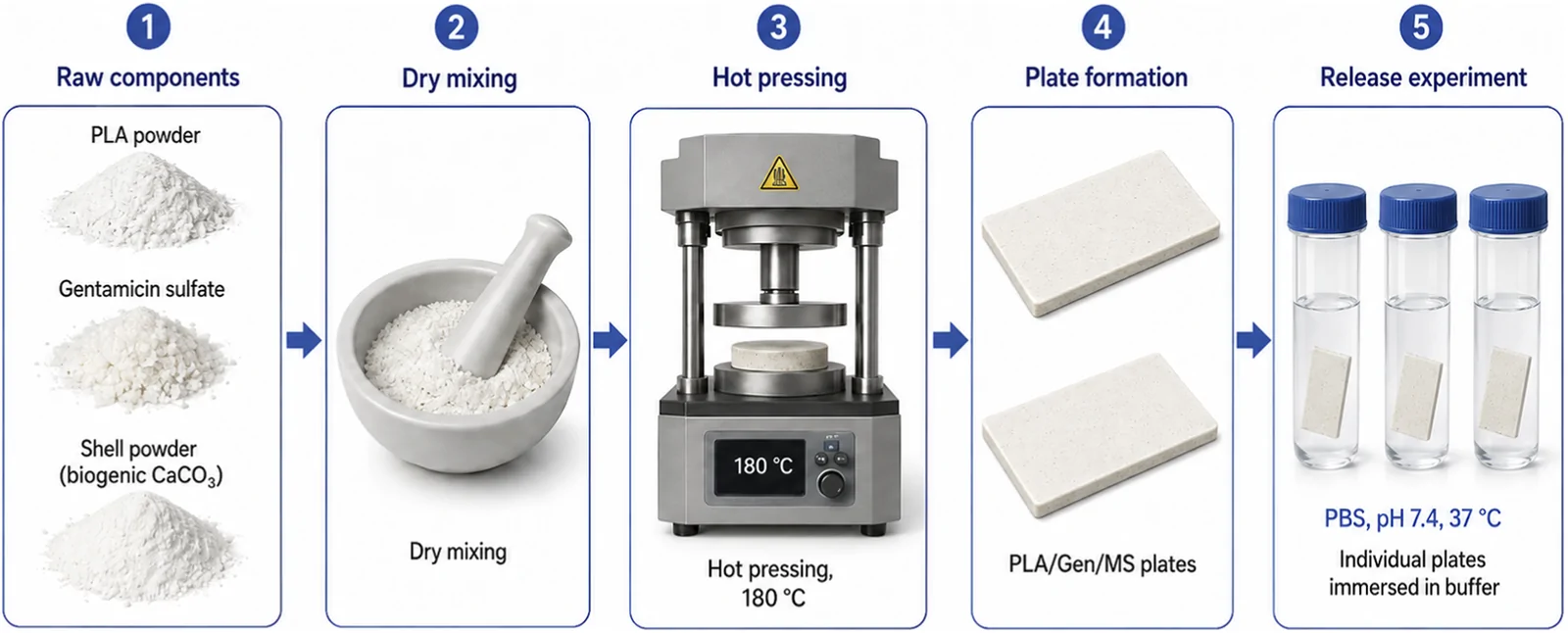
Schematic illustration of PLA-GM-MS plate preparation and the gentamicin release experiment. This schematic was prepared with the assistance of ChatGPT (OpenAI, San Francisco, CA, USA).

### 2.4 FTIR spectroscopy

Fourier-transform infrared (FTIR) spectroscopy was used to characterize the mollusk shell-derived mineral powder and representative hot-pressed PLA-GM and PLA-GM-MS20 matrices. Spectra were recorded using a Nicolet iS10 spectrometer (Thermo Fisher Scientific, Waltham, MA, USA) over the mid-infrared range of 4,000–400 cm^-1^.

The FTIR spectra were evaluated to identify the characteristic absorption regions associated with the shell-derived carbonate-containing mineral phase, the PLA matrix, and gentamicin sulfate. Comparative analysis of the PLA-GM and PLA-GM-MS20 spectra was additionally performed to assess the spectral contribution of the mineral phase after its incorporation into the polymer matrix.

### 2.5 SEM/EDS characterization

The morphology of the mollusk shell-derived mineral powder and the microstructure of representative PLA-GM-MS matrices were examined by scanning electron microscopy (SEM) using a Helios 5 CX dual-beam SEM/FIB system (Thermo Fisher Scientific, Waltham, MA, USA).

SEM imaging of the shell-derived powder was performed at different magnifications to characterize particle morphology and particle dimensions. Representative particle dimensions were determined directly from the SEM micrographs.

For characterization of the hot-pressed matrices, two representative formulations were selected for qualitative microstructural comparison: PLA-GM, serving as the mineral-free reference matrix, and PLA-GM-MS20, representing a shell-containing composite with an intermediate MS content. SEM imaging was used to assess surface morphology, matrix continuity, and the spatial organization of the mineral-containing microstructure.

Energy-dispersive X-ray spectroscopy (EDS) mapping was additionally performed for PLA-GM-MS20 to evaluate the spatial distribution of the principal elements within the analyzed region. Elemental maps of carbon (C), oxygen (O), and calcium (Ca) were obtained to characterize the polymer–mineral microstructure and to verify the spatial distribution of the shell-derived Ca-containing phase within the composite matrix.

### 2.6 Water uptake measurement

Water uptake of the PLA-GM-MS composite matrices was evaluated in phosphate-buffered saline (PBS, pH 7.4). Formulations containing 0, 10, 20, 30, and 40 wt% mollusk shell powder were investigated. Before the experiment, the specimens were dried to constant mass and their initial dry mass (*m*_*0*_ *)* was recorded.

The specimens were then completely immersed in PBS (pH 7.4) and incubated at 37 °C. Measurements were performed after 1, 2, 3, 6, and 12 days of incubation. At each time point, the specimens were removed from the medium, excess surface liquid was gently removed using filter paper, and the specimens were immediately weighed to determine the wet mass (*m*_*t*_).

Water uptake was calculated according to the following equation:

$$\text{WU }\left(\%\right)=\left[\left(m_{t}-m_{0}\right)\;/\;m_{0}\right]\times 100$$

where m_0_ is the initial dry mass of the specimen (g), and m_t_ is the specimen mass after incubation in PBS at the corresponding time point (g).

Five specimens were analyzed for each formulation (n = 5). The results are presented as mean ± standard deviation (SD).

### 2.7 Gentamicin release study

The release kinetics of gentamicin sulfate from the PLA-GM-MS composite matrices were investigated in phosphate-buffered saline (PBS, pH 7.4) at 37 °C. Individual film specimens were placed in sealed tubes containing PBS at a ratio of 10 mL per 1 g of sample. Incubation was performed in a thermostat under static conditions without additional agitation.

Samples were collected after 1, 2, 4, 8, and 16 days of incubation. An additional late-stage measurement was performed at day 32, extending the experimental observation period beyond the primary 1–16 days kinetic series, to assess whether gentamicin release continued after the apparent plateau observed by day 16. The day-32 measurement was used as a confirmatory late-stage endpoint and was not included in the primary kinetic analysis. At each time point, a 0.1 mL aliquot was withdrawn from each tube for analysis, and the removed volume was replaced with an equivalent volume of fresh PBS. The replacement of sampled medium with fresh buffer was taken into account when calculating the cumulative amount of gentamicin released.

The concentration of gentamicin sulfate in the collected samples was determined spectrophotometrically after derivatization with ascorbic acid. Gentamicin release was evaluated separately for each of the three independently hot-pressed specimens of every formulation (n = 3). Each specimen was incubated in an individual tube throughout the release study.

### 2.8 Quantification of gentamicin sulfate

Gentamicin sulfate was quantified using a spectrophotometric method after derivatization with ascorbic acid in dimethyl sulfoxide (DMSO), according to a previously described procedure (Khrustaleva et al., 2026a). For analysis, 0.1 mL of the test solution was transferred into a heat-resistant volumetric tube, mixed with 0.5 mL of ascorbic acid solution in DMSO at a concentration of 0.025 g/mL, and brought to a final volume of 5 mL with DMSO. The resulting solutions were heated in a water bath for 45 min and subsequently cooled at room temperature for 15 min.

The absorbance of the derivatized solutions was measured at 365 and 510 nm using a PD-303 spectrophotometer (Apel Co., Japan). Quantitative determination of gentamicin sulfate was based on the absorbance measured at 510 nm.

Calibration was performed at five gentamicin concentration levels (20, 40, 60, 80, and 100 µg/mL), with three replicate measurements at each concentration. The calibration data were fitted by linear regression constrained through the origin, yielding the equation

$$A_{510}=0.00550C,$$

where *A*_*510*_ is the absorbance at 510 nm and *C* is the gentamicin concentration in µg/mL. The calibration showed excellent linearity over the investigated concentration range (^*R2*^ = 0.9994). The relative standard deviation (RSD) of replicate absorbance measurements ranged from 1.40% to 10.18%, with the highest variability observed at the lowest calibration level (20 µg/mL). The individual calibration measurements are provided in Supplementary Table S1, and the corresponding calibration curve is shown in Supplementary Figure S1.

The limits of detection (LOD) and quantification (LOQ) were estimated from the residual standard deviation of the zero-intercept calibration regression and the calibration slope according to *LOD = 3.3σ/S* and *LOQ = 10σ/S*. The resulting *LOD* and *LOQ* were 5.44 and 16.48 µg/mL, respectively.

Potential interference from the composite matrix was evaluated using gentamicin-free PLA-based matrices processed and analyzed under the same conditions as the drug-containing specimens. No substantial absorbance attributable to the drug-free matrix was observed at 510 nm, indicating that the matrix components did not materially interfere with gentamicin quantification under the applied analytical conditions.

### 2.9 Release data processing and geometric normalization

The cumulative amount of gentamicin sulfate released from each sample at the specified time points was denoted as *M*_*t*_ *,* expressed in mg, where *t* is the incubation time. Absolute *M*_*t*_ values were used to construct individual release profiles because they reflect the actual amount of antibiotic transferred from each sample into the buffer medium.

Because the investigated composite plates differed in mass, thickness, and calculated surface area, the release data were additionally normalized to the sample mass and to the calculated total area of the two major film surfaces for subsequent quantitative comparison.

Mass-normalized release was calculated as follows:

$$Q_{m}=M_{t}\;/\;m,$$

where *Q*_*m*_ is the amount of gentamicin released normalized to the sample mass, mg/g; *M*_*t*_ is the cumulative amount of gentamicin released at time *t*, mg; and *m* is the sample mass, g.

Cumulative gentamicin release was also expressed as a percentage of the nominal gentamicin loading of each specimen:

$$\text{Rt }\left(\%\right)=M_{t}\;/\;\left(0.05\;\times m\times \;1000\right)\times 100,$$

where *M*_*t*_ is the cumulative amount of gentamicin released at time *t*, mg; *m* is the specimen mass, g; and 0.05 corresponds to the nominal gentamicin content of 5 wt%.

For geometric normalization, the total area of the two major surfaces of each film specimen *(A)* was calculated. The sample volume was determined as follows:

V=m / ρ,

where *V* is the calculated sample volume, mm^3^; *m* is the sample mass, mg; and *ρ* is the calculated density of the composite material, mg/mm^3^. The density used for this geometric estimate was a composition-based theoretical value derived from the constituent densities and the nominal formulation composition.

The calculated total area of the two major surfaces was determined using the following equation:

A=2V / h,

where *A* is the calculated total area of the two major surfaces of the sample, mm^2^; *V* is the calculated sample volume, mm^3^; and *h* is the mean sample thickness, mm. The factor of two accounts for the two major surfaces of the film specimen. The specimens were treated as thin plate-like matrices, for which the two major planar surfaces represent the principal release surfaces and the contribution of the edges is comparatively small when the lateral dimensions substantially exceed specimen thickness (Kalosakas and Martini, 2015).

Area-normalized release was calculated as follows:

$$Q_{a}=\left(M_{t}\;/\;A\right)\times 1000,$$

where *Q*_*a*_ is the amount of gentamicin released normalized to the calculated surface area, μg/mm^2^. The factor of 1,000 was used to convert mg to μg.

For quantitative comparison of the investigated compositions, *M*_*16*_ *, Q*_*m*_ *, Q*_*a*_ and *R*_*16*_ values obtained on day 16 of the experiment were used.

### 2.10 Kinetic modeling of gentamicin release

Gentamicin release kinetics were evaluated at the individual-specimen level using area-normalized cumulative release data. For each specimen, the cumulative amount of gentamicin released at time *t*, *M*_t_, was normalized to the calculated surface area of the two major specimen surfaces according to the equation:

$$Q_{t}=\left(M_{t}\;/\;A\right)\times 1000,$$

where *Q*_t_ is the area-normalized cumulative release (µg/mm^2^), *M*_t_ is the corrected cumulative amount of gentamicin released (mg), and *A* is the calculated specimen surface area (mm^2^). The factor 1,000 converts mg to µg.

For analysis of the early release stage, the simplified Higuchi relationship was applied as an empirical description of the square-root-of-time release behavior (Siepmann and Peppas, 2011):

$$Q_{t}=K_{H}\times t^{1/2},$$

where *K*_*H*_ is the apparent Higuchi release constant (µg·mm^-2^·h^-1/2^) and *t* is time in hours. A common fitting interval of 0–4 days was used for all specimens, comprising the origin and the experimental measurements obtained at 1, 2, and 4 days. Linear regression was constrained through the origin. The goodness of fit was characterized using the coefficient of determination (*R* ^2^) and root mean square error (RMSE).

To compare the square-root-of-time relationship with a constant-rate release process, the same 0–4-day dataset was additionally fitted using the zero-order model:

$$Q_{t}=k_{0}\times t,$$

where *k*_0_ is the zero-order release constant (µg·mm^-2^·h^-1^). The relative performance of the Higuchi and zero-order models was evaluated using the corrected Akaike information criterion (AICc), with the model yielding the lower AICc considered to provide the better empirical description of the data.

Because the experimental release profiles extended beyond the initial release stage and progressively approached a plateau, the complete 0–16-day datasets were additionally evaluated using a first-order asymptotic model:

$$Q_{t}=Q_{\infty }\times \left[1-\text{exp}\left(-k_{1}\times \;t\right)\right],$$

where $Q_{\infty }$ is the fitted asymptotic area-normalized release (µg/mm^2^) and *k*_1_ is the first-order release constant (h^-1^). Higuchi, zero-order, and first-order fits of the complete release profiles were compared using *R* ^2^, RMSE, and AICc. Residuals were examined to assess systematic deviations between the experimental and fitted values.

The applicability of the Korsmeyer–Peppas and Hixson–Crowell models was also considered. These models were not included in the final comparison because their underlying requirements were not adequately matched by the present release dataset. Reliable Korsmeyer–Peppas analysis requires a sufficiently resolved early fractional-release region for estimation of the exponent n, whereas the Hixson–Crowell model relies on assumptions related to a defined initial drug amount and geometry-dependent depletion. Applying these models under the present conditions would therefore provide parameters of limited physical reliability rather than strengthen the kinetic interpretation.

The relationship between the apparent Higuchi release constant *K*_*H*_ and mollusk shell powder content was evaluated by simple linear regression using the individual specimen values:

$$K_{H}=\beta_{0}+\beta_{1}\times W$$

where *W* is the mollusk shell powder content (wt%), *β*_*0*_ is the regression intercept, and *β*_*1*_ is the regression coefficient for shell powder content. Statistical significance was accepted at *p* < 0.05.

The robustness of the composition-dependent kinetic trend with respect to the geometric normalization was additionally evaluated using an area-independent sensitivity analysis, as described in Section 2.12.

### 2.11 Antibacterial activity of hot-pressed composite matrices

The antibacterial activity of the hot-pressed PLA-GM-MS composite matrices was evaluated using the agar diffusion (disk-diffusion) method against the reference bacterial strains *Staphylococcus aureus* ATCC 29213 and *Escherichia coli* ATCC 25922. Bacterial suspensions were adjusted to a 0.5 McFarland standard and uniformly inoculated onto Mueller–Hinton agar plates using the lawn culture method.

Hot-pressed composite specimens containing 5 wt% gentamicin sulfate and 0, 10, 20, 30, or 40 wt% shell-derived mineral phase were placed on the surface of the inoculated agar. Standard gentamicin disks (10 µg/disk) were used as a positive control. Three specimens were tested for each composite formulation (n = 3).

The plates were incubated at 37 °C for 24 h. After incubation, the diameters of the bacterial growth inhibition zones were measured in millimeters using a caliper (Stayer, Pinto, Madrid, Spain; resolution 0.01 mm). The results are presented as mean ± SEM.

### 2.12 Statistical analysis

For the release and kinetic analyses, three independently fabricated specimens were analyzed for each formulation (n = 3). Individual values were retained for descriptive presentation because of the observed intersample variability. Because specimen thickness varied among the hot-pressed matrices and may independently influence gentamicin release through the effective diffusion path length, the association between mineral-phase content and gentamicin release was additionally evaluated using multiple linear regression while accounting for specimen thickness.

Mass-normalized gentamicin release at day 16 (*Q*_*m*_ *,* mg/g) was used as the dependent variable, while mollusk shell powder content (wt%) and specimen thickness (mm) were included as continuous independent variables. *Q*_*m*_ was selected for this analysis because, unlike the area-normalized parameter *Q*_*a*_ *,* it does not mathematically incorporate specimen thickness through the calculation of surface area.

As an additional area-independent sensitivity analysis, early-stage release was also expressed on a mass-normalized basis as *Q*_*m,t*_ *= M*_*t*_ */m.* For each specimen, the 0–4-day data were fitted to *Q*_*m,t*_ *=K*_*H,m*_ *t* ^*1/2*^, using the same fitting interval and origin constraint as in the primary Higuchi analysis. The resulting mass-normalized apparent Higuchi constant *K*_*H,m*_ (mg·g^-1^·h^-1/2^) was then analyzed by multiple linear regression with shell powder content and experimentally measured specimen thickness as continuous independent predictors. This analysis was used to determine whether the composition-dependent kinetic trend remained evident when calculated density and surface area were excluded entirely.

Regression assumptions were evaluated using residual diagnostics. Linearity of the association with shell powder content was assessed by comparison with a model including a quadratic term and with a model treating shell powder content as a categorical factor. Residual normality was evaluated using the Shapiro–Wilk test, homoscedasticity using the Breusch–Pagan test, and collinearity between predictors using variance inflation factors (VIF). Influential observations were assessed using Cook’s distance. The stability of the regression coefficients was further evaluated using leave-one-out sensitivity analysis, in which the model was refitted after sequential exclusion of each individual specimen.

Regression coefficients (*B*), 95% confidence intervals, coefficients of determination (*R* ^*2*^ and adjusted *R* ^*2*^ *),* and *p* values were calculated. Statistical significance was defined as *p* < 0.05.

## 3 Results

### 3.1 Physicochemical and morphological characterization of the mollusk shell powder

The mollusk shell powder used as the mineral filler was characterized by complementary spectroscopic, elemental, and microscopic methods (Figure 2).

**FIGURE 2:**
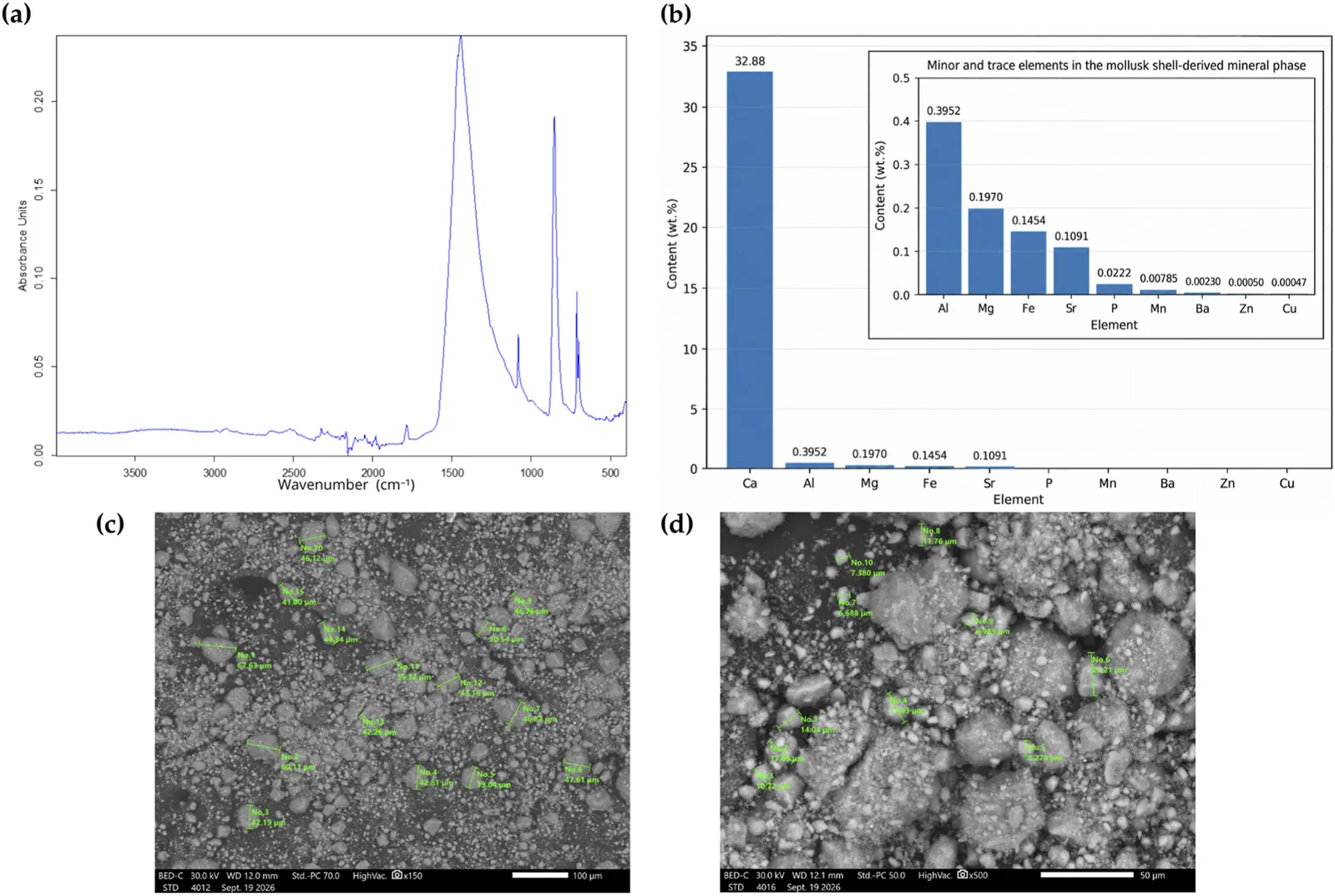
Physicochemical and morphological characterization of the mollusk shell-derived mineral powder used as the mineral filler. **(a)** FTIR spectrum of the shell powder; **(b)** elemental composition demonstrating the predominance of Ca, with an inset showing the minor and trace elements on an expanded scale; **(c)** SEM micrograph at ×150 and **(d)** SEM micrograph at ×500, showing irregularly shaped mineral particles with heterogeneous dimensions.

FTIR analysis revealed a spectrum dominated by characteristic carbonate vibrations (Figure 2a). The most intense absorption occurred in the approximately 1,450 cm^-1^ region and was assigned to the asymmetric stretching vibration, ν_3_, of CO_3_^2-^. Additional diagnostic carbonate bands were observed near approximately 860 and 710 cm^-1^, corresponding to the ν_2_ out-of-plane and ν_4_ in-plane bending vibrations, respectively. The overall FTIR profile was characteristic of a carbonate-rich mineral material.

Elemental analysis independently confirmed the strongly Ca-rich character of the shell-derived mineral phase (Figure 2b). Calcium was the predominant detected element, accounting for 32.88 wt% of the analyzed material. The principal minor elements were Al (0.3952 wt%), Mg (0.1970 wt%), Fe (0.1454 wt%), and Sr (0.1091 wt%), whereas P, Mn, Ba, Zn, and Cu were present at substantially lower levels. The marked predominance of Ca, together with the diagnostic carbonate bands observed by FTIR, provides convergent evidence that the filler represents a Ca-rich carbonate-containing biogenic mineral phase.

SEM examination further demonstrated the heterogeneous particulate morphology of the shell powder (Figures 2c,d). At lower magnification (Figure 2c), the powder consisted of irregularly shaped mineral fragments accompanied by numerous smaller particles. Representative dimensions of the larger particles measured within the examined field were approximately 36–68 µm. Higher-magnification imaging (Figure 2d) revealed a substantial population of smaller particles, with representative dimensions of approximately 6–23 μm, together with irregular aggregates and fragments of varying morphology. These observations demonstrate that the milled shell material possessed a broad range of particle dimensions and a distinctly heterogeneous particulate architecture.

Taken together, FTIR, elemental analysis, and SEM provided mutually consistent chemical and morphological characterization of the filler used in this study. The shell-derived material was identified as a Ca-rich, carbonate-containing biogenic mineral powder with irregular particulate morphology and a broad range of particle dimensions observed by SEM, providing a defined physicochemical basis for its subsequent incorporation into the PLA-GM-MS composite matrices.

### 3.2 Physical and geometric characteristics of the PLA-GM-MS composite matrices

To investigate the release kinetics of gentamicin sulfate, a series of PLA-based composite matrices containing 0, 10, 20, 30, and 40 wt% mollusk shell powder was prepared at a constant gentamicin sulfate loading of 5 wt%. The samples were designated as follows: PLA-GM, a matrix without mollusk shell powder; and PLA-GM-MS10, PLA-GM-MS20, PLA-GM-MS30, and PLA-GM-MS40, composite matrices containing 10, 20, 30, and 40 wt% mollusk shell powder, respectively. The main physical and geometric characteristics of the investigated samples are presented in Table 2.

**TABLE 2:** Physical and geometric characteristics of the PLA-GM-MS composite matrices used for normalization of gentamicin release.

Sample No.	Sample	Shell powder, wt%	Mass, g	Thickness, mm	Density, mg/mm^3^	A, mm^2^
1	PLA-GM	0	0.873	0.40	1.1364	3,842
2	PLA-GM	0	0.916	0.45	1.1364	3,584
3	PLA-GM	0	0.962	0.35	1.1364	4,839
4	PLA-GM-MS10	10	0.919	0.35	1.2728	4,126
5	PLA-GM-MS10	10	1.048	0.40	1.2728	4,118
6	PLA-GM-MS10	10	0.929	0.70	1.2728	2086
7	PLA-GM-MS20	20	1.078	0.40	1.4091	3,827
8	PLA-GM-MS20	20	1.002	0.50	1.4091	2,844
9	PLA-GM-MS20	20	0.908	0.80	1.4091	1,610
10	PLA-GM-MS30	30	1.050	0.55	1.5455	2,471
11	PLA-GM-MS30	30	1.112	0.60	1.5455	2,399
12	PLA-GM-MS30	30	0.889	0.85	1.5455	1,354
13	PLA-GM-MS40	40	0.792	0.50	1.6818	1883
14	PLA-GM-MS40	40	1.032	0.55	1.6818	2,232
15	PLA-GM-MS40	40	1.153	0.75	1.6818	1827

A is the calculated total area of the two major surfaces of the sample.

Because drug release from film-like matrix systems is determined not only by material composition, but also by the amount of loaded drug, diffusion path length, and contact area with the aqueous medium, the mass, mean thickness, calculated density, and total area of the two major surfaces (*A*) were determined for each matrix. These parameters were used as initial values for subsequent normalization of the release data and comparative evaluation of the transport characteristics of composites with different compositions. Because specimen thickness may also influence release through the effective diffusion path length, its contribution was additionally considered in the subsequent statistical analysis.

The mass of the investigated plates ranged from 0.792 to 1.153 g, whereas their thickness varied from 0.35 to 0.85 mm. As a result, the calculated surface area available for contact with the buffer medium differed by more than threefold, with A values ranging from 1,354 to 4,839 mm^2^. The highest surface area values were characteristic of thinner samples, whereas an increase in thickness at comparable sample mass was associated with a decrease in surface area. This factor is important for the interpretation of kinetic data, because samples with different contact areas may exhibit different absolute amounts of released antibiotic even when the matrix composition is identical.

Increasing the content of mollusk shell powder led to the expected increase in the calculated density of the composite matrices. For PLA-GM samples without mineral filler, the calculated density was 1.1364 mg/mm^3^, whereas for PLA-GM-MS40 it reached 1.6818 mg/mm^3^. This increase in density reflects the higher fraction of the inorganic phase in the composite and further indicates the need to account for both composition and sample geometry when comparing gentamicin release.

All prepared samples retained their integrity during incubation in phosphate-buffered saline and were suitable for subsequent analysis of gentamicin release. Considering the observed differences in mass, thickness, and calculated surface area, further evaluation was performed not only using the absolute amount of released antibiotic, but also using parameters normalized to sample mass and calculated surface area. This approach allowed differences in sample mass and calculated contact area to be taken into account in the subsequent comparison of gentamicin release, while the independent contribution of specimen thickness was evaluated statistically.

### 3.3 Physicochemical and microstructural characterization of representative PLA-GM-MS matrices

FTIR spectra of the hot-pressed PLA-GM and PLA-GM-MS20 matrices are shown in Figure 3a.

**FIGURE 3:**
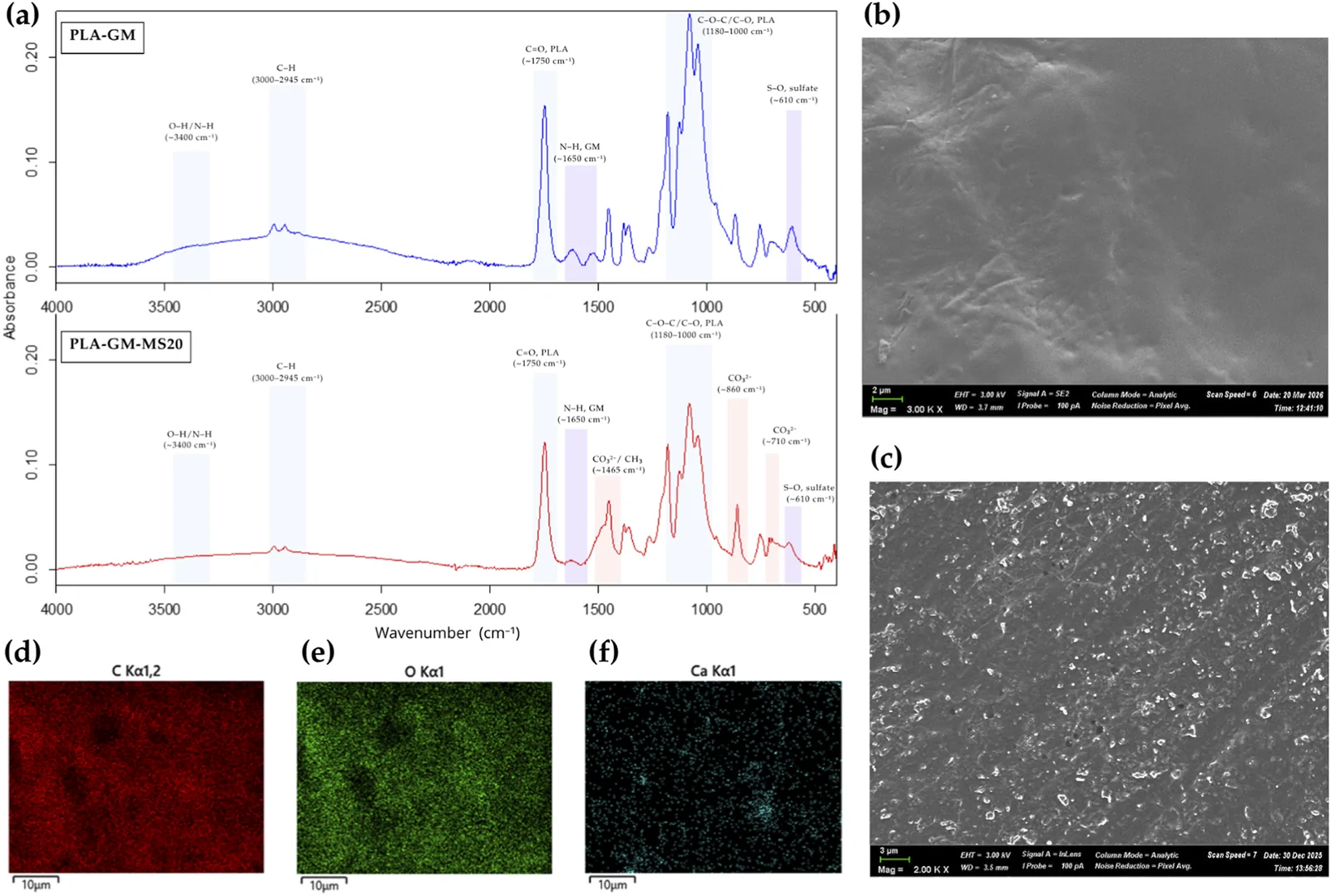
Physicochemical and microstructural characterization of PLA-GM and PLA-GM-MS20 matrices. **(a)** FTIR spectra of PLA-GM and PLA-GM-MS20; SEM images of **(b)** PLA-GM and **(c)** PLA-GM-MS20; EDS elemental maps of **(d)** C, **(e)** O, and **(f)** Ca for PLA-GM-MS20.

Both formulations exhibited the characteristic spectral features of the PLA matrix, including the ester carbonyl stretching band at approximately 1750 cm^-1^, C–H stretching bands in the 3,000–2,945 cm^-1^ region, and intense C–O–C/C–O stretching vibrations in the 1,180–1,000 cm^-1^ fingerprint region. The preservation of these characteristic bands in both formulations indicated that the principal chemical structure of the PLA matrix was retained after hot pressing.

Spectral features associated with gentamicin sulfate were also observed in both matrices. The contribution near approximately 1,650 cm^-1^ was consistent with N–H deformation vibrations of gentamicin, while the low-wavenumber feature near 610 cm^-1^ was assigned to sulfate-associated S–O vibrations. Because gentamicin was present at 5 wt% and several of its characteristic vibrations overlap with the more intense PLA bands, its contribution was expressed primarily as overlapping features rather than as a set of isolated high-intensity peaks.

Incorporation of 20 wt% mollusk shell powder introduced additional carbonate-related features into the PLA-GM-MS20 spectrum. The absorption around 1,465 cm^-1^ represented an overlapping contribution of the ν_3_ asymmetric stretching vibration of CO_3_^2-^ and CH_3_ deformation vibrations of PLA. More diagnostic carbonate bands were observed at approximately 860 and 710 cm^-1^, corresponding to the ν_2_ out-of-plane and ν_4_ in-plane bending vibrations of CO_3_^2-^, respectively. Thus, comparison of the two spectra demonstrated the retention of the principal PLA- and gentamicin-associated spectral features together with the appearance of characteristic carbonate contributions after incorporation of the shell-derived mineral phase. No new major absorption bands indicative of pronounced chemical transformation of the PLA matrix were observed.

Complementary SEM examination revealed distinct differences in the microstructural organization of PLA-GM and PLA-GM-MS20 (Figures 3b,c). The PLA-GM specimen exhibited a dense and continuous surface with relatively low structural contrast and no apparent interconnected micron-scale pore network within the examined region. Incorporation of 20 wt% MS substantially increased the local microstructural heterogeneity while preserving the overall compact character of the hot-pressed matrix. Numerous high-contrast domains were distributed throughout the PLA-GM-MS20 surface, producing a heterogeneous polymer–mineral architecture without the development of an extensive interconnected micron-scale pore network.

EDS elemental mapping of PLA-GM-MS20 (Figures 3d–f) provided compositional confirmation of this heterogeneous microstructure. Carbon and oxygen were broadly distributed throughout the analyzed region, whereas calcium was detected as multiple spatially dispersed Ca-rich domains. The Ca distribution was consistent with the mineral-associated features observed by SEM, confirming incorporation and spatial dispersion of the shell-derived Ca-rich phase within the continuous PLA matrix.

Overall, the combined FTIR and SEM/EDS characterization demonstrated that the hot-pressed PLA-GM-MS20 composite retained the principal chemical signatures of PLA and gentamicin sulfate while incorporating a spatially dispersed carbonate-rich mineral phase. The resulting material exhibited a compact but compositionally and microstructurally heterogeneous polymer–mineral architecture, with dispersed Ca-rich domains and extensive polymer–mineral interfacial contact.

### 3.4 Water uptake of PLA-GM-MS matrices

Water uptake was evaluated to determine whether incorporation of the shell-derived mineral phase altered the interaction of the hot-pressed composite matrices with the aqueous medium. A composition-dependent increase in water uptake was observed across the investigated formulations (Figure 4).

**FIGURE 4:**
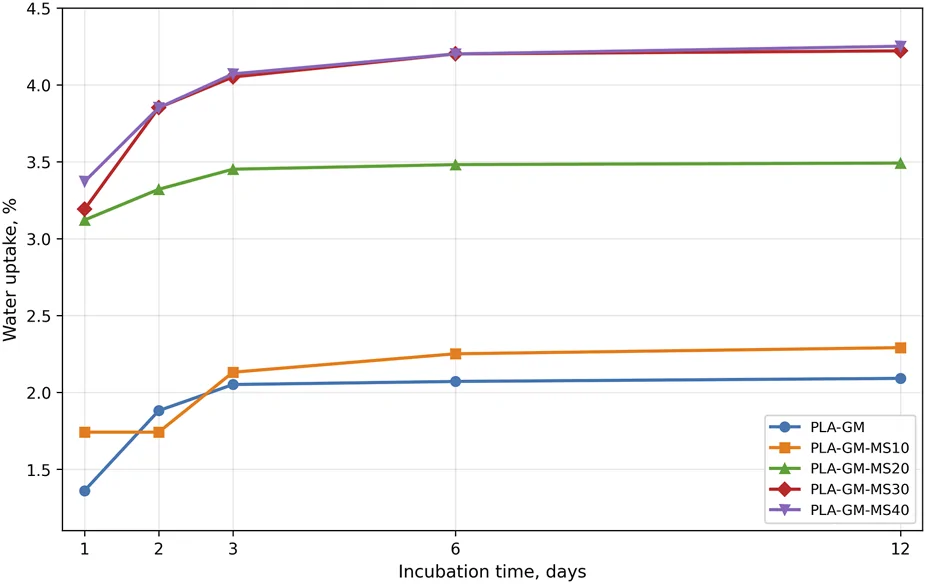
Water uptake of PLA-GM-MS composite matrices during incubation in phosphate-buffered saline (PBS, pH 7.4). Water uptake was determined for matrices containing 0, 10, 20, 30, and 40 wt% mollusk shell powder over 12 days at 37 °C.

For the PLA‐GM matrix without shell powder, water uptake increased from 1.36 ± 0.47% on day 1 to 2.09 ± 0.54% on day 12. The PLA-GM-MS10 formulation showed only a modest increase, reaching 2.29 ± 0.63% by day 12. More pronounced water uptake was observed for matrices containing 20–40 wt% MS. At day 12, the corresponding values were 3.49 ± 0.59% for PLA-GM-MS20, 4.22 ± 0.57% for PLA-GM-MS30, and 4.25 ± 0.61% for PLA-GM-MS40.

For all formulations, the largest increase occurred during the early incubation period, whereas only minor additional changes were observed between days 6 and 12. At the highest mineral-phase contents, 30 and 40 wt% MS, the water-uptake values approached a similar plateau of approximately 4.2%.

Overall, increasing the shell-derived mineral-phase content was associated with greater water uptake by the PLA-GM-MS matrices, with the most pronounced differences observed between the low- and high-mineral formulations. These data provide direct experimental evidence that the mineral-rich matrices exhibited greater interaction with the aqueous medium during incubation.

### 3.5 Gentamicin release profiles from PLA-GM-MS composite matrices

Individual cumulative release profiles of gentamicin sulfate from the PLA-GM-MS composite matrices are presented in Figure 5. Samples containing 0, 10, 20, 30, and 40 wt% mollusk shell powder were investigated at a constant nominal gentamicin sulfate loading of 5 wt%. The primary release profiles were evaluated in phosphate-buffered saline at pH 7.4 and 37 °C over a 16-day period, with an additional late-stage measurement performed at day 32. In this section, the results are presented as absolute values, expressed in mg, allowing evaluation of the experimentally determined cumulative amount of antibiotic transferred from each individual sample into the buffer medium. The specimen-level absorbance values, calculated gentamicin concentrations, medium-replacement corrections, and corrected cumulative release values underlying the profiles shown in Figure 5 are provided in Supplementary Table S2.

**FIGURE 5:**
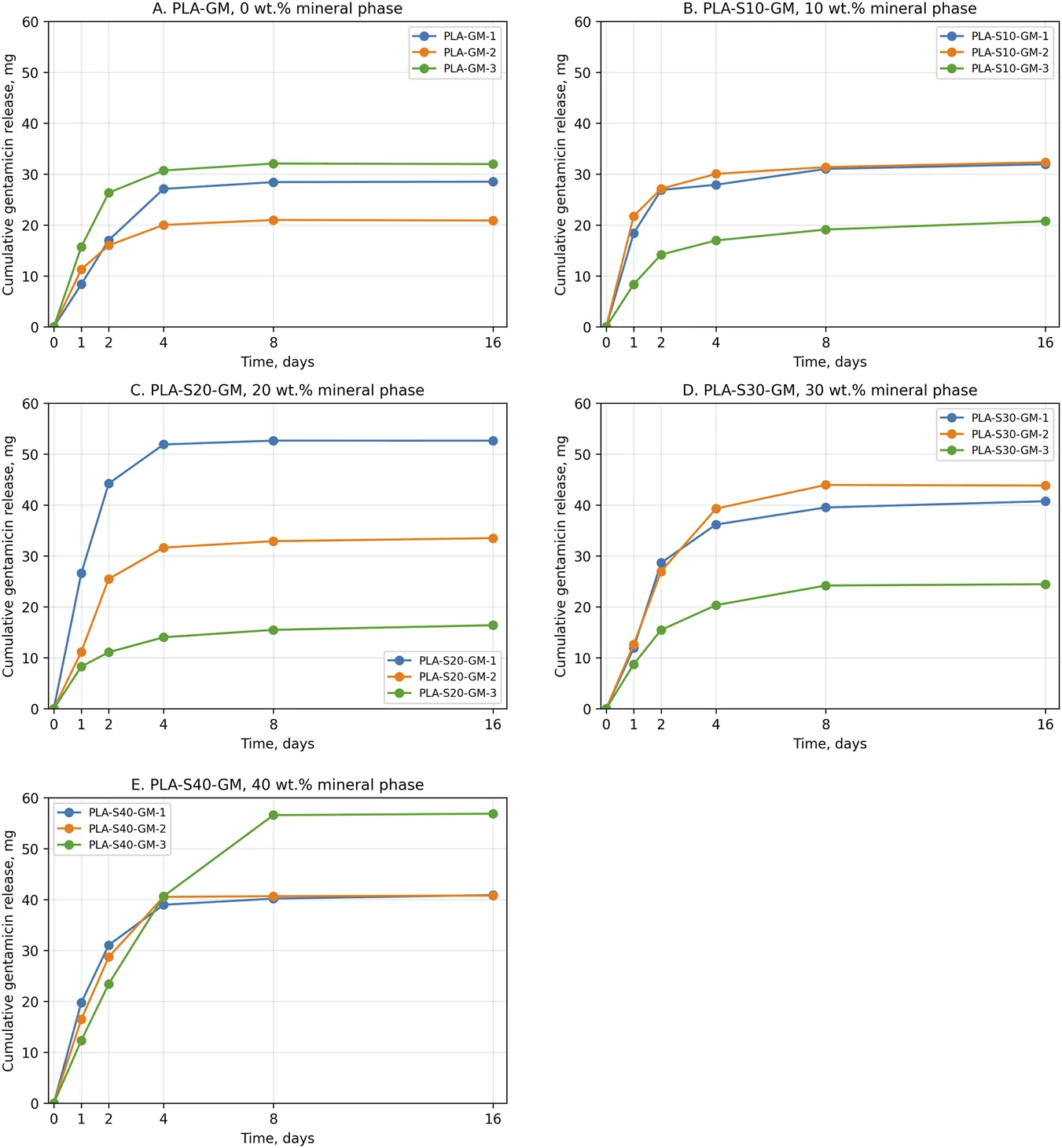
Individual cumulative release profiles of gentamicin sulfate from PLA-GM-MS composite matrices with different mollusk shell powder contents: **(A)** PLA-GM, 0 wt%; **(B)** PLA-GM-MS10, 10 wt%; **(C)** PLA-GM-MS20, 20 wt%; **(D)** PLA-GM-MS30, 30 wt%; and **(E)** PLA-GM-MS40, 40 wt%. Values are presented as absolute amounts, mg. Incubation conditions: phosphate-buffered saline, pH 7.4, 37 °C.

All investigated compositions exhibited a similar biphasic release behavior. The most intensive gentamicin release occurred during the initial stage of the experiment, mainly within the first 1–4 days. Thereafter, the rate of antibiotic accumulation in the buffer medium decreased, and most individual profiles approached a plateau between days 8 and 16. Release monitoring was extended to day 32 to verify whether the apparent plateau observed by day 16 persisted over a longer observation period. No meaningful further increase in gentamicin release was detected at day 32 compared with day 16, indicating that the plateau persisted during the subsequent 16-day period. Thus, although release was experimentally followed for 32 days, the systematically sampled 1–16 days interval was retained for the primary comparative and kinetic analyses, while day 32 served as a confirmatory late-stage endpoint. Thus, regardless of the mineral phase content, gentamicin release from the hot-pressed PLA-GM-MS matrices was characterized by an initial stage of rapid antibiotic release followed by a gradual slowing of the process.

For PLA-GM matrices without mollusk shell powder, the cumulative amount of gentamicin released by day 16 ranged from 20.89 to 31.97 mg. The incorporation of 10 wt% mineral phase did not lead to an obvious increase in absolute release: for PLA-GM-MS10, the values ranged from 20.76 to 32.34 mg. The widest variation among individual samples was observed for PLA-GM-MS20, for which the day-16 cumulative release ranged from 16.40 to 52.64 mg. For PLA-GM-MS30 composites, the amount of gentamicin transferred into the solution by day 16 was in the range of 24.45–43.84 mg.

The highest overall range of day-16 cumulative release was observed for the PLA-GM-MS40 composites, in which the amount of gentamicin detected in the buffer medium ranged from 40.79 to 56.87 mg. Although one PLA-GM-MS20 specimen also exhibited a high cumulative release value of 52.64 mg, all three PLA-GM-MS40 specimens showed comparatively high day-16 release values (40.79–56.87 mg). At the same time, the individual curves within each group differed in the rate of initial release and in the level at which they approached a plateau, highlighting the importance of analyzing not only averaged data, but also individual release profiles.

When cumulative gentamicin release was expressed as a percentage of the nominal 5 wt% drug loading, the same overall composition-dependent trend was observed. The day-16 percentage release values are summarized in Table 3. Overall, higher mollusk shell powder contents were associated with a greater fraction of nominally incorporated gentamicin released by day 16. Because percentage release was calculated relative to the nominal 5 wt% gentamicin loading rather than to an experimentally determined post-processing drug content, individual values may slightly exceed 100%; such values should therefore be interpreted as apparent cumulative release relative to the nominal loading rather than as absolute recovery.

**TABLE 3:** Day-16 cumulative gentamicin release from PLA-GM-MS matrices after normalization to sample mass and calculated surface area.

Sample No.	Sample	Shell powder, wt%	*M*_*16*_ *,* mg	*Q*_*m*_ *,* mg/g	*R*_*16*_ *, %*	*A,* mm^2^	*Q*_*a*_ *,* μg/mm^2^
1	PLA-GM	0	28.52	32.67	65.33	3,842	7.42
2	PLA-GM	0	20.89	22.79	45.59	3,584	5.83
3	PLA-GM	0	31.97	33.23	66.45	4,839	6.61
4	PLA-GM-MS10	10	31.94	34.75	69.50	4,126	7.74
5	PLA-GM-MS10	10	32.34	30.85	61.70	4,118	7.85
6	PLA-GM-MS10	10	20.76	22.34	44.68	2086	9.95
7	PLA-GM-MS20	20	52.64	48.81	97.63	3,827	13.76
8	PLA-GM-MS20	20	33.49	33.44	66.87	2,844	11.78
9	PLA-GM-MS20	20	16.40	18.06	36.13	1,610	10.18
10	PLA-GM-MS30	30	40.76	38.81	77.63	2,471	16.49
11	PLA-GM-MS30	30	43.84	39.41	78.82	2,399	18.27
12	PLA-GM-MS30	30	24.45	27.49	54.99	1,354	18.06
13	PLA-GM-MS40	40	40.90	51.68	103.36	1883	21.72
14	PLA-GM-MS40	40	40.79	39.53	79.05	2,232	18.28
15	PLA-GM-MS40	40	56.87	49.34	98.69	1827	31.13

M_16_ is the cumulative amount of gentamicin released by day 16; Q_m_ is the mass-normalized release calculated as M_16_/m; R_16_ is the cumulative release expressed as a percentage of the nominal 5 wt% gentamicin loading; A is the calculated total area of the two major surfaces of the specimen; and Q_a_ is the area-normalized release calculated as M_16_/A × 1,000.

Overall, analysis of the absolute release profiles showed that all investigated matrices exhibited a biphasic gentamicin release pattern, whereas the highest day-16 cumulative release values as a group were observed for composites containing 40 wt% mineral phase. However, the absolute amount of released antibiotic depended on the individual geometric and mass characteristics of the samples, including mass, thickness, and contact area with the buffer medium. Therefore, to compare the transport properties of PLA-GM-MS composites with different compositions, further analysis was performed using parameters normalized to sample mass and calculated surface area.

### 3.6 Effect of mass and geometric normalization on the evaluation of gentamicin release

Because the investigated composite matrices differed in mass, thickness, and calculated surface area, the absolute values of cumulative release did not allow the effect of material composition to be clearly distinguished from the contribution of individual sample geometry. Therefore, for a more appropriate comparison of composites with different compositions, the day-16 cumulative amount of released gentamicin was additionally normalized to the sample mass and to the calculated total area of the two major surfaces. Because specimen thickness may also influence drug release through the effective diffusion path length, its contribution was additionally evaluated using multivariable statistical analysis, as described below. The summarized *M*_*16*_ *, Q*_*m*_ *,* and *Q*_*a*_ values are presented in Table 3.

After normalization to sample mass, *Q*_*m*_ values for PLA-GM matrices without mollusk shell powder ranged from 22.79 to 33.23 mg/g. For PLA-GM-MS10 composites, *Q*_*m*_ ranged from 22.34 to 34.75 mg/g, which was comparable to that of PLA-GM. In the PLA-GM-MS20 group, *Q*_*m*_ values varied over a wider range, from 18.06 to 48.81 mg/g. For PLA-GM-MS30, the day-16 *Q*_*m*_ values were 27.49–39.41 mg/g, whereas the highest values were observed for PLA-GM-MS40, ranging from 39.53 to 51.68 mg/g.

A more pronounced dependence on mineral phase content was observed when area-normalized release, *Q*_*a*_ *,* was analyzed. For PLA-GM, *Q*_*a*_ values ranged from 5.83 to 7.42 μg/mm^2^. With the incorporation of 10 wt% mollusk shell powder, these values increased to 7.74–9.95 μg/mm^2^. For composites containing 20 and 30 wt% mineral phase, *Q*_*a*_ values were 10.18–13.76 and 16.49–18.27 μg/mm^2^, respectively. The highest range of area-normalized release was obtained for PLA-GM-MS40, reaching 18.28–31.13 μg/mm^2^.

Thus, compared with the absolute M_16_ values and the mass-normalized parameter *Q*_*m*_ *,* the *Q*_*a*_ parameter showed a clearer descriptive relationship with mollusk shell powder content. Within the investigated compositional range, increasing the mineral phase fraction from 0 to 40 wt% was accompanied by a progressive increase in day-16 area-normalized release. This trend was particularly evident for compositions containing 20–40 wt% mollusk shell powder. Individual *Q*_*a*_ values and group medians are shown in Figure 6.

**FIGURE 6:**
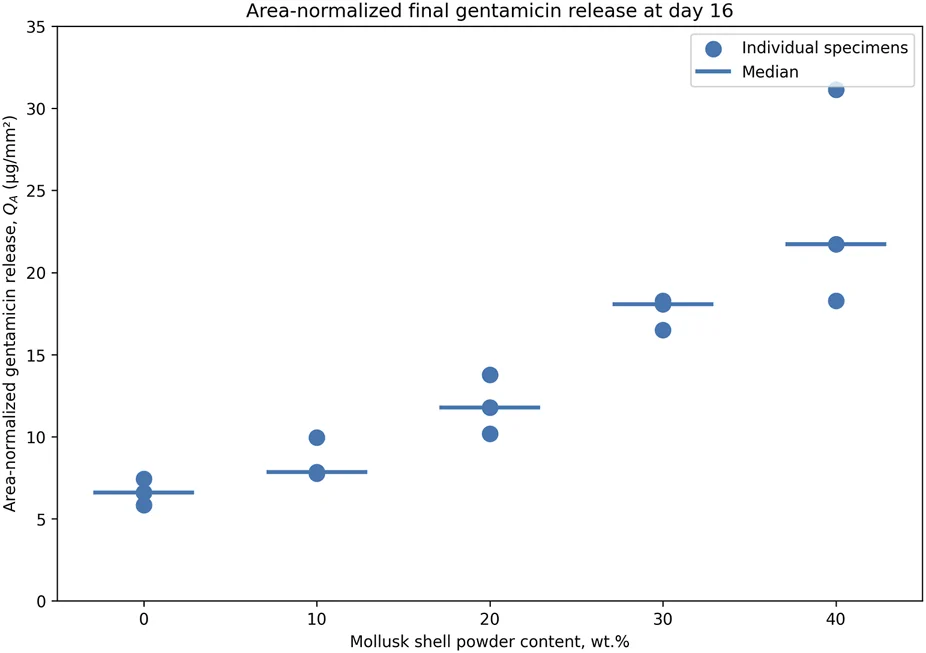
Effect of mollusk shell powder content on the area-normalized gentamicin release from PLA-GM-MS composite matrices at day 16. Values are presented as *Q*_*a*_ *,* μg/mm^2^, calculated as the amount of gentamicin released by day 16 divided by the calculated total area of the two major surfaces of the sample (*A*). Points correspond to individual samples; horizontal lines indicate group medians.

To evaluate the composition–release relationship independently of the calculated surface area and density, mass-normalized release at day 16 *Q*_*m*_ was used for multivariable analysis. Because specimen thickness varied between individual matrices and may independently affect drug release through the effective diffusion path length, the contributions of mineral-phase content and thickness were examined using multiple linear regression. Unlike *Q*_*a*_ *, Q*_*m*_ does not contain calculated surface area or density. Mollusk shell powder content (*W*, wt%) and specimen thickness (*h*, mm) were included as continuous independent predictors. The corresponding regression coefficients and their statistical significance are summarized in Table 4.

**TABLE 4:** Multiple linear regression analysis of factors associated with mass-normalized gentamicin release at day 16.

Predictor	B	95% CI	p
Intercept	46.211	35.432–56.989	<0.001
Mollusk shell powder content, wt%	0.671	0.422–0.919	0.000075
Specimen thickness, mm	−45.539	−67.656 to −23.423	0.000744

Model *R* ^2^ = 0.757; adjusted *R* ^2^ = 0.716; overall model p = 0.00021; n = 15.

The resulting regression equation was:

$$Q_{m}=46.211+0.671W-45.539h.$$

The overall regression model was statistically significant (*R* ^*2*^ = 0.757, adjusted *R* ^*2*^ = 0.716, p = 0.00021; Table 4). Mollusk shell powder content remained significantly and positively associated with mass-normalized gentamicin release after adjustment for specimen thickness, whereas specimen thickness showed an independent negative association with release. At constant specimen thickness, an increase of 10 wt% in mollusk shell powder content corresponded to an estimated increase of approximately 6.71 mg/g in mass-normalized gentamicin release by day 16.

Regression diagnostics supported the use of the linear model. Inclusion of a quadratic term for shell powder content did not significantly improve the model (*p* = 0.472), and treating the five shell-content levels as a categorical factor provided no significant improvement over the continuous linear representation (*F* = 0.204, *p* = 0.891). The residuals showed no evidence of departure from normality (Shapiro–Wilk *W* = 0.971, *p* = 0.878) or heteroscedasticity (Breusch–Pagan *p* = 0.261). Collinearity between shell powder content and specimen thickness was low, with a variance inflation factor (VIF) of 1.37 for both predictors. The corresponding regression diagnostic plots are presented in Supplementary Figure S2.

Influence diagnostics identified specimen 15 as the most influential observation (Cook’s distance = 0.493). Leave-one-out sensitivity analysis nevertheless showed that the observed composition–release association was not dependent on this or any other individual specimen. Across all 15 leave-one-out models, the regression coefficient for shell powder content remained positive (*B* = 0.606–0.771) and statistically significant *(p* = 0.000028–0.000567). When specimen 15 was excluded specifically, shell powder content remained significantly associated with mass-normalized gentamicin release (*B* = 0.606, *p* = 0.000048; *R* ^*2*^ = 0.831). Thus, no single specimen determined the positive association between shell powder content and gentamicin release. The complete leave-one-out sensitivity analysis is provided in Supplementary Table S3.

The robustness of the composition-dependent relationship was further evaluated using an additional area-independent analysis of the early release stage. For each specimen, cumulative release over 0–4 days was normalized only to specimen mass, and the resulting mass-normalized square-root-of-time slope, *K*_*H,m*_, was analyzed as a function of shell powder content and specimen thickness. This sensitivity model was also statistically significant (*R* ^*2*^ = 0.836, adjusted *R* ^*2*^ = 0.809, overall *p* = 1.91 × 10^−5^). Shell powder content remained a significant positive predictor (*B* = 0.0603, 95% CI 0.0398–0.0809, *p* = 0.000034), whereas specimen thickness showed a significant negative association (*B* = −6.024, 95% CI −7.851 to −4.197, *p* = 0.000011). Thus, the composition-dependent increase was retained for the early-stage release behavior even when calculated density and surface area were excluded entirely from the analysis.

These findings indicate that specimen thickness contributed independently to the observed variability in gentamicin release, consistent with the expected influence of diffusion path length in compact matrices. Importantly, however, the positive association between mollusk shell powder content and gentamicin release remained statistically significant after adjustment for thickness. Moreover, the same composition-dependent trend was observed in both the day-16 cumulative release and the early-stage kinetic sensitivity analysis when neither calculated density nor calculated surface area was used. Therefore, the observed relationship cannot be attributed solely to the geometric assumptions underlying the area-normalized parameter *Q*_*a*_.

Taken together, the descriptive area-normalized results and the complementary mass-normalized analyses provide a consistent picture in which increasing mollusk shell powder content is associated with enhanced gentamicin release from PLA-GM-MS matrices. The persistence of this relationship after complete exclusion of calculated density and surface area strengthens the evidence for a composition-dependent effect on gentamicin release behavior and provides the basis for the subsequent kinetic analysis of the investigated matrices.

### 3.7 Kinetic modeling of gentamicin release

To quantitatively compare gentamicin release kinetics among the PLA-GM-MS composite matrices, the simplified Higuchi relationship was applied to the early stage of the release profiles. A common fitting interval of 0–4 days was used for all specimens, corresponding to the initial release stage before the pronounced transition toward the plateau observed at later time points. The resulting apparent Higuchi release constants, *K*_*H*_, were used to compare the early-stage area-normalized release behavior of the different PLA-GM-MS formulations.

The early release profiles showed good agreement with the square-root-of-time relationship. The *R* ^2^ values for the individual specimens ranged from 0.9143 to 0.9902, with a mean value of 0.9614, while RMSE values ranged from 0.237 to 2.082 µg/mm^2^ (Table 5).

**TABLE 5:** Parameters of the simplified Higuchi fits for early-stage area-normalized gentamicin release from individual PLA-GM-MS specimens.

Sample No.	Composition	Shell powder, wt%	K_H_, µg·mm^-2^·h^-1/2^	R^2^	RMSE, µg/mm^2^
1	PLA-GM	0	0.658	0.9465	0.606
2	PLA-GM	0	0.602	0.9872	0.237
3	PLA-GM	0	0.689	0.9737	0.396
4	PLA-GM-MS10	10	0.793	0.9193	0.771
5	PLA-GM-MS10	10	0.851	0.9143	0.837
6	PLA-GM-MS10	10	0.873	0.9788	0.454
7	PLA-GM-MS20	20	1.470	0.9756	0.815
8	PLA-GM-MS20	20	1.133	0.9489	0.981
9	PLA-GM-MS20	20	0.942	0.9840	0.411
10	PLA-GM-MS30	30	1.473	0.9413	1.386
11	PLA-GM-MS30	30	1.572	0.9536	1.328
12	PLA-GM-MS30	30	1.535	0.9861	0.664
13	PLA-GM-MS40	40	2.194	0.9902	0.769
14	PLA-GM-MS40	40	1.805	0.9861	0.794
15	PLA-GM-MS40	40	2.024	0.9353	2.082

K_H_, was obtained from linear fitting of the area-normalized cumulative release, Q_t_, against the square root of time over the common 0–4-day interval (0, 1, 2, and 4 days), with the regression constrained through the origin. *R* ^2^ is the coefficient of determination and RMSE, is the root mean square error of the fit.

A progressive increase in *K*_*H*_ was observed with increasing mollusk shell powder content. For PLA-GM matrices without shell powder, *K*_*H*_ ranged from 0.602 to 0.689 µg·mm^-2^·h^-1/2^, with a mean value of 0.650 ± 0.044 µg·mm^-2^·h^-1/2^. The mean *K*_*H*_ increased to 0.839 ± 0.041 µg·mm^-2^·h^-1/2^ for PLA-GM-MS10, 1.182 ± 0.268 µg·mm^-2^·h^-1/2^ for PLA-GM-MS20, 1.527 ± 0.050 µg·mm^-2^·h^-1/2^ for PLA-GM-MS30, and 2.007 ± 0.195 µg·mm^-2^·h^-1/2^ for PLA-GM-MS40. Thus, the mean apparent Higuchi release constant increased by approximately 3.1-fold between the formulations containing 0 and 40 wt% mollusk shell powder.

To compare the square-root-of-time relationship with a constant-rate release model, the same 0–4-day data were additionally analyzed using the zero-order model. Comparison based on the corrected Akaike information criterion (AICc) favored the Higuchi relationship for 13 of the 15 individual specimens. The mean *R* ^2^ value was 0.961 for the Higuchi fits compared with 0.798 for the zero-order fits, indicating that the square-root-of-time relationship provided the better description of the early release stage for most specimens. The individual Higuchi fits and corresponding residual plots are shown in Supplementary Figures S3, S4, respectively, while the specimen-level comparison of the Higuchi and zero-order models is summarized in Supplementary Table S4.

The relationship between mollusk shell powder content and the apparent Higuchi release constant was further evaluated using the individual specimen values (Figure 7). A strong positive linear relationship was obtained:

$$K_{H}=0.5604+0.03403W$$

$$R^{2}=0.9185,p\;<\;0.0001,$$

where *W* is the mollusk shell powder content (wt%). The regression coefficient for shell powder content was 0.03403 µg·mm^-2^·h^-1/2^ per wt% (95% CI: 0.02796–0.04010), demonstrating a systematic increase in the early-stage release constant across the investigated composition range.

**FIGURE 7:**
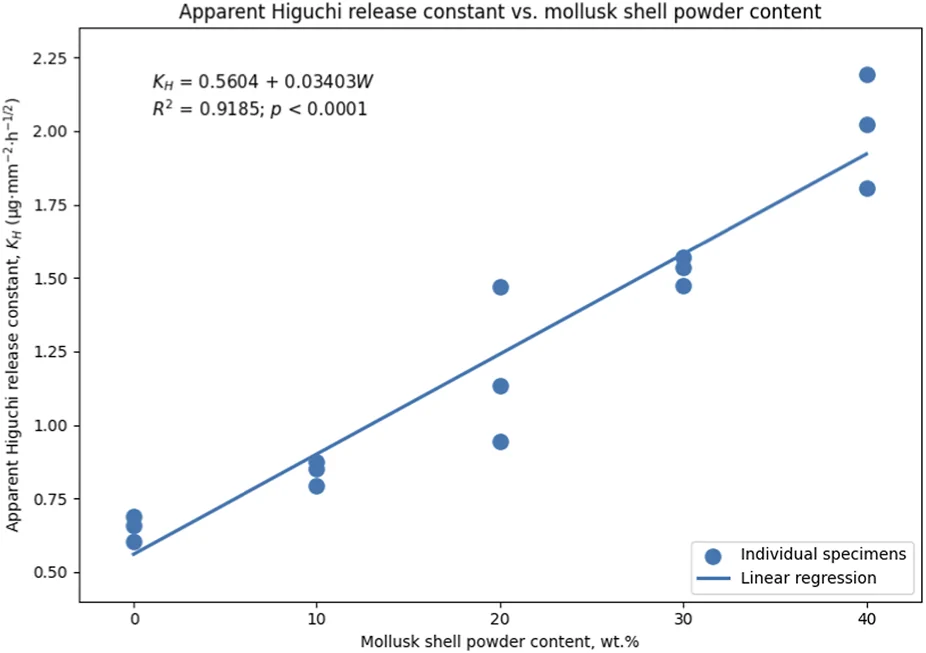
Relationship between mollusk shell powder content and the apparent Higuchi release constant *(K*_*H*_ *)* for PLA-GM-MS composite matrices.

Because the complete release profiles progressively approached a plateau at later time points, the 0–16-day datasets were additionally evaluated using alternative release models. Among the models examined, the first-order asymptotic model provided the best overall description of the complete release profiles, with *R* ^2^ values ranging from 0.9786 to 0.9995 and the lowest AICc for all 15 specimens. The individual first-order fits for the complete 0–16-day release profiles are shown in Supplementary Figure S5, while the specimen-level fitting parameters and model-comparison statistics are summarized in Supplementary Table S5. This behavior is consistent with the experimentally observed release pattern, characterized by a relatively rapid initial stage followed by a gradual approach to a plateau.

Overall, the kinetic analysis demonstrated a clear composition-dependent change in gentamicin release behavior. The progressive increase in *K*_*H*_ with increasing mollusk shell powder content was consistent with the increase in area-normalized cumulative release observed at day 16, indicating that incorporation of the shell-derived mineral phase systematically enhanced gentamicin release from the PLA-based composite matrices.

### 3.8 Antibacterial activity of hot-pressed composite matrices

The antibacterial activity of the hot-pressed composite matrices containing 5 wt% gentamicin sulfate and 0–40 wt% shell-derived mineral phase was evaluated by the agar diffusion method against *Staphylococcus aureus* and *Escherichia coli*. All investigated formulations produced clearly defined growth inhibition zones against both test microorganisms (Table 6; Figure 8).

**TABLE 6:** Antibacterial activity of hot-pressed PLA-GM-MScomposite matrices with different contents of shell-derived mineral phase.

Formulation	*S. aureus*, ATCC 29213, inhibition zone, mm (mean ± SEM)	*E. coli*, ATCC 25922, inhibition zone, mm (mean ± SEM)
PLA-GM	22.63 ± 0.38	23.75 ± 0.25
PLA-GM-MS10	22.63 ± 0.38	22.00 ± 0.58
PLA-GM-MS20	23.00 ± 0.41	22.50 ± 0.29
PLA-GM-MS30	23.00 ± 0.41	23.00 ± 0.41
PLA-GM-MS40	23.50 ± 0.54	22.75 ± 0.63
Gentamicin control	20.33 ± 0.21	17.92 ± 0.33
PLA-MS	-	-
PLA	-	-

**FIGURE 8:**
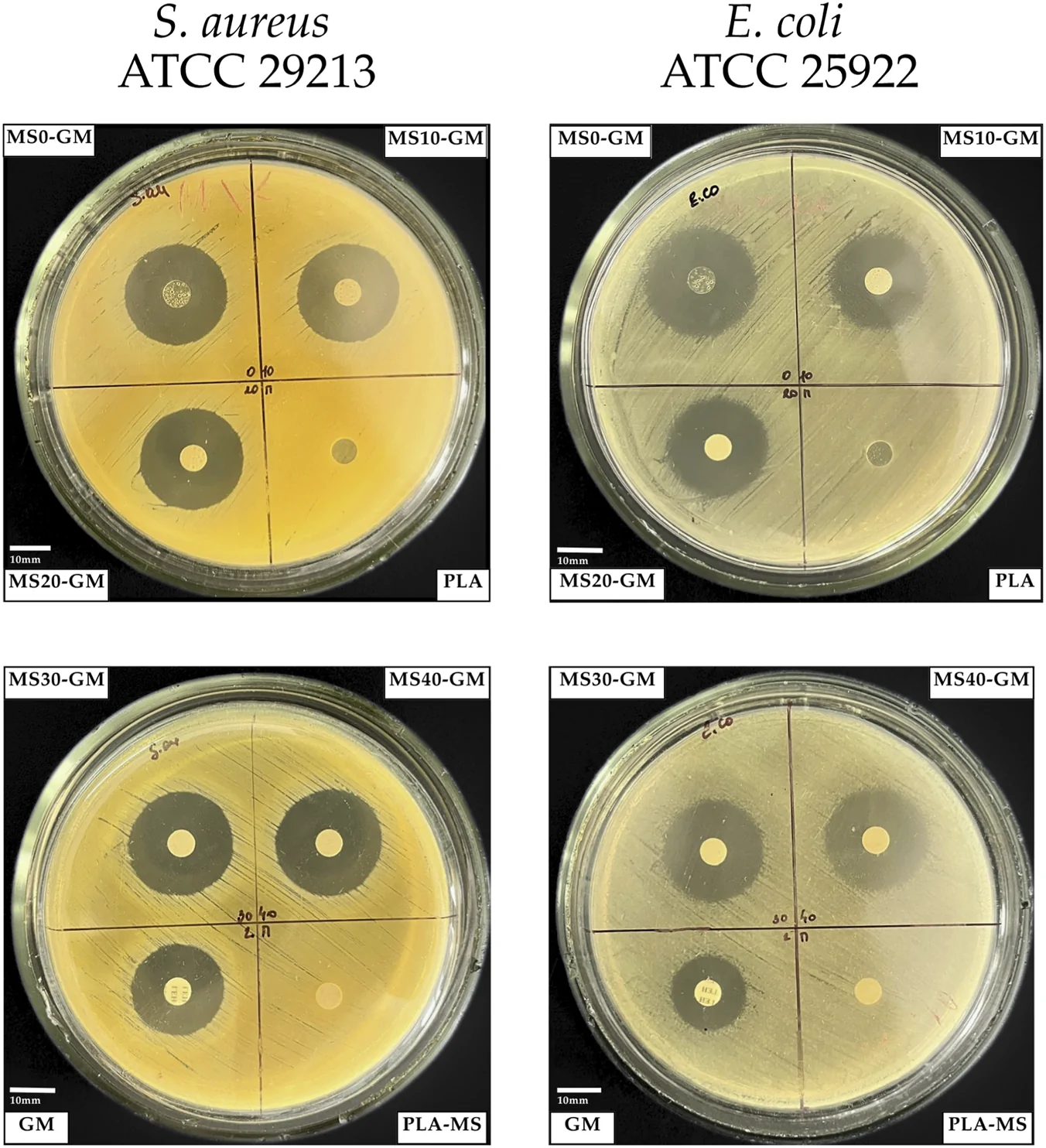
Representative agar diffusion assay showing antibacterial activity of hot-pressed PLA-GM-MS matrices against *Staphylococcus aureus* ATCC 29213 and *Escherichia coli* ATCC 25922. MS0-GM, MS10-GM, MS20-GM, MS30-GM, and MS40-GM denote PLA matrices containing 5 wt% gentamicin sulfate and 0, 10, 20, 30, and 40 wt% mollusk shell powder, respectively. PLA, neat PLA without gentamicin or shell powder; PLA-MS, PLA containing mollusk shell powder without gentamicin; GM, gentamicin control.

For *S. aureus*, the mean inhibition-zone diameters ranged within a narrow interval from 22.63 ± 0.38 to 23.50 ± 0.54 mm, whereas for *E. coli*, they ranged from 22.00 ± 0.58 to 23.75 ± 0.25 mm. Thus, pronounced antibacterial activity after hot pressing at 180 °C was confirmed for all investigated formulations containing 0–40 wt% mineral phase.

The similar inhibition-zone diameters observed among the investigated formulations are consistent with the characteristics of the disk-diffusion method, since the size of the inhibition zone is determined not only by the amount of antibacterial agent released from the specimen, but also by its release rate from the matrix, subsequent diffusion through the agar medium, specimen geometry, and the susceptibility of the test microorganism. Therefore, the disk-diffusion results should primarily be interpreted as functional confirmation that gentamicin retained its antibacterial activity after hot pressing, rather than as a quantitative measure of differences in gentamicin release kinetics among the formulations.

Overall, the results confirm that the hot-pressed PLA-GM-MS composite matrices retained pronounced antibacterial activity against both *S. aureus* and *E. coli* after processing at 180 °C. Together with the quantitative data on gentamicin release in PBS, these findings demonstrate the preservation of antibacterial functionality of the investigated composites following thermal processing.

## 4 Discussion

The key challenge in developing biodegradable systems for the local delivery of hydrophilic antibiotics lies not merely in incorporating the drug into a polymer matrix, but in controlling its transport accessibility (Chang et al., 2006; Pisani et al., 2019). PLA, a widely used biodegradable polyester, has limited hydrophilicity, which restricts the penetration of aqueous media into compact matrices and, consequently, the release of water-soluble drugs (Pisani et al., 2019; Yoo and Won, 2020; Fredenberg et al., 2011). Conventional strategies for addressing this limitation—including modification of the polymer chemistry, use of copolymers, incorporation of pore-forming agents, and fabrication of microspheres, electrospun fibers, or highly porous scaffolds—can be effective, but they are technologically more complex and make it difficult to separate the contributions of carrier chemistry, morphology, and architecture to the observed release profile (Yoo and Won, 2020; Kanak et al., 2023; Fredenberg et al., 2011). In the present study, we propose an alternative approach in which gentamicin sulfate transport is regulated by varying the content of a shell-derived biogenic CaCO_3_-containing phase, namely, mollusk shell powder, within a compact hot-pressed PLA matrix, without chemical modification of the polymer and without increasing the nominal antibiotic loading.

The additional antibacterial evaluation showed that all hot-pressed PLA-GM-MS formulations retained activity against both *Staphylococcus aureus* and *Escherichia coli*, indicating that neither thermal processing at 180 °C nor incorporation of the shell-derived mineral phase over the investigated concentration range abolished the antibacterial functionality of the gentamicin-containing matrices. This finding is consistent with our previous studies demonstrating preservation of antimicrobial activity in thermally processed gentamicin-containing PLA composites, including hot-pressed PLA/hydroxyapatite/gentamicin systems (Khrustaleva et al., 2026a; Khrustaleva et al., 2026c). Because inhibition-zone formation is influenced by diffusion through the agar medium, specimen characteristics, and microorganism susceptibility, these results should be interpreted as formulation-specific confirmation of retained antibacterial activity rather than as a quantitative measure of gentamicin release.

From a technological perspective, the development of such systems lies at the interface between pharmaceutical processing and materials science: alongside the intensification of drug compound production (Khrustalev, 2009; Khrustalev et al., 2022), increasing importance is being placed on controlling their incorporation into biodegradable carriers and their subsequent local release.

The main experimental finding of this study is that increasing the content of the biogenic CaCO_3_ phase in PLA-GM-MS composites was accompanied by a progressive increase in both area-normalized gentamicin release (*Q*_*a*_ *)* and the apparent Higuchi release constant, *K*_*H*_. Importantly, the composition-dependent trend remained evident after accounting for specimen geometry. Area normalization revealed a progressive increase in day-16 cumulative gentamicin release with increasing mineral-phase content, while multiple linear regression further showed that mollusk shell powder content remained significantly and positively associated with mass-normalized release after adjustment for specimen thickness. At the same time, specimen thickness showed an independent negative association with release, consistent with the expected influence of a longer transport path in compact matrices. Taken together, these findings indicate that the observed increase in gentamicin release cannot be explained solely by geometric differences among the specimens and support an independent contribution of composite composition to release behavior.

The mechanistic interpretation of this composition-dependent effect is strengthened by the complementary microstructural and physicochemical characterization performed in the present study. SEM examination showed that the mineral-free PLA-GM matrix retained a compact and continuous morphology, whereas incorporation of 20 wt% shell-derived mineral powder produced a distinctly more heterogeneous polymer–mineral microstructure containing numerous spatially distributed mineral-associated domains. EDS mapping further demonstrated the presence of dispersed Ca-rich regions within the continuous PLA matrix. Importantly, no extensive interconnected micron-scale pore network was apparent in the examined SEM fields. Thus, the increase in gentamicin release observed for the mineral-containing formulations is not readily explained by conversion of the compact matrices into highly porous structures.

Instead, incorporation of the mineral phase appears to alter the internal polymer–mineral organization and the interaction of the matrix with the aqueous medium. The water-uptake measurements provide direct physicochemical support for this interpretation: increasing MS content was accompanied by progressively greater water uptake, with the mineral-rich formulations exhibiting the highest values. This trend indicates enhanced interaction of the composite matrices with PBS and greater aqueous accessibility as the mineral fraction increased. In combination with the SEM/EDS observations, these findings support a mechanism in which dispersed mineral domains and the associated PLA/mineral interfacial regions facilitate water penetration and increase the accessibility of incorporated gentamicin for dissolution and subsequent diffusion. As the mineral-phase content increases, the number and spatial extent of such polymer–mineral interfaces are also expected to increase, consistent with the observed increases in both Q_a_ and the early-stage apparent Higuchi release constant, KH. A schematic representation of the proposed mechanism by which the content of the biogenic CaCO_3_-containing phase regulates gentamicin transport is shown in Figure 9.

**FIGURE 9:**
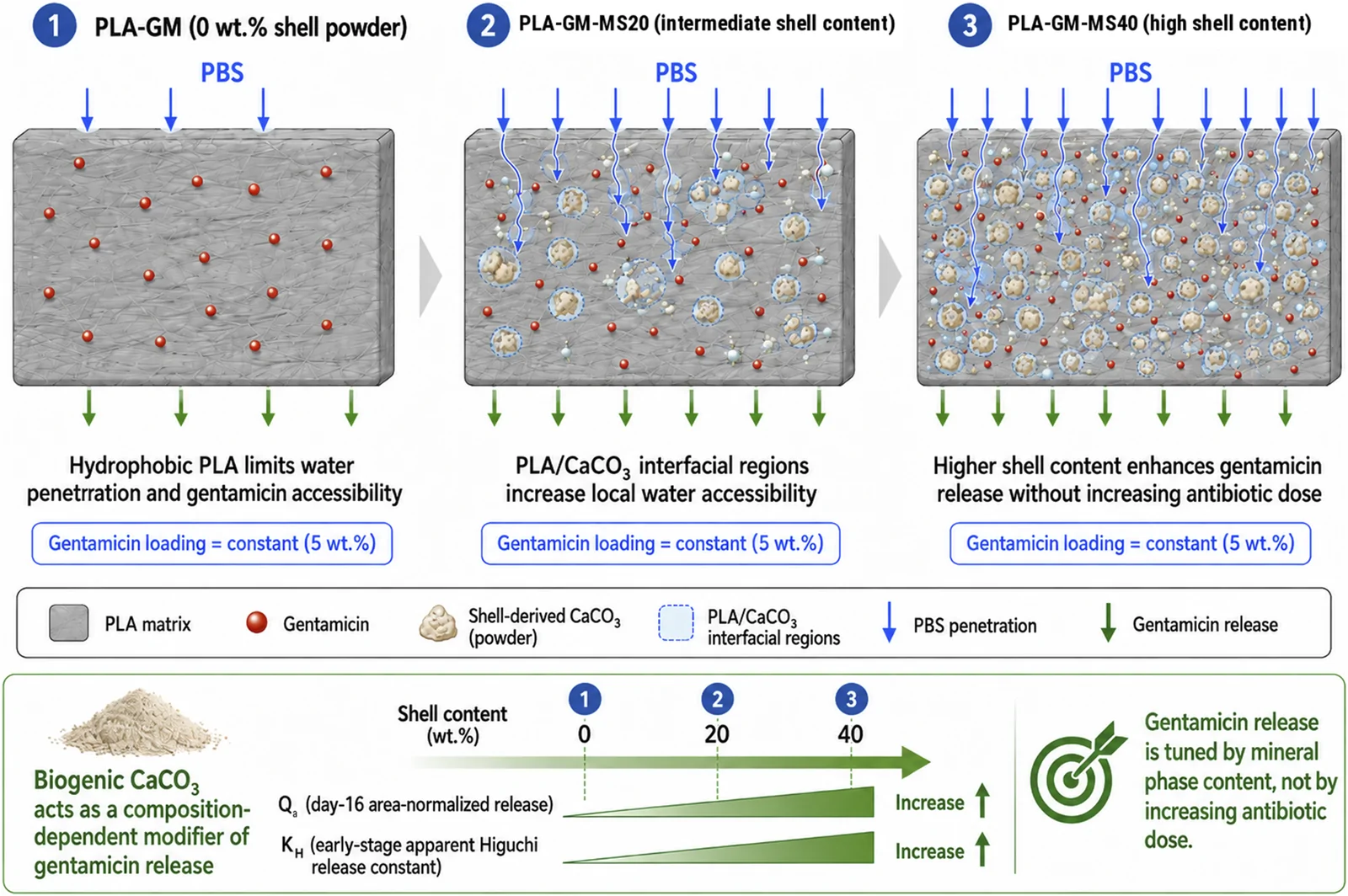
Conceptual interpretation of the composition-dependent modulation of gentamicin release in hot-pressed PLA-GM-MS composites. Increasing the content of the biogenic CaCO_3_-containing phase may enhance aqueous accessibility of the matrix and increase the contribution of PLA/CaCO_3_ interfacial regions, thereby facilitating gentamicin dissolution and diffusion and contributing to the observed increases in area-normalized cumulative release Q_a_ and the apparent Higuchi release constant *K*_*H*_. This schematic illustration was prepared by the authors with the assistance of ChatGPT (OpenAI, San Francisco, CA, USA).

Literature data support the importance of calcium-containing components in the interaction of PLA-based materials with aqueous environments. Donate et al. showed that CaCO_3_ coating of 3D-printed PLA scaffolds increases surface hydrophilicity and affects the behavior of the material in an aqueous medium (Donate et al., 2023). However, in that study, CaCO_3_ was used as a surface coating, whereas in the present work the mineral phase is distributed throughout the bulk of a dense matrix and influences transport across the entire composite. Related approaches to regulating gentamicin release using calcium-containing mineral phases have been described for PLA/nHAp nanocomposites and PLGA/nHA microspheres (Kanak et al., 2023). However, in these systems, release kinetics are largely determined by high specific surface area, porosity, and microspherical or fibrous morphology. In contrast, the present SEM observations indicate that the PLA-GM-MS matrices remained compact rather than developing an extensive interconnected micron-scale porous network. This supports the interpretation that mineral–polymer interfacial organization and enhanced aqueous accessibility can contribute to antibiotic transport even in compact hot-pressed matrices.

The physicochemical nature of the shell-derived filler is also relevant to the observed behavior. In the present study, complementary FTIR, ICP-AES, and SEM characterization established the starting shell powder as a Ca-rich, carbonate-containing biogenic mineral material with irregular particulate morphology. FTIR showed the characteristic carbonate absorption features, while ICP-AES demonstrated that Ca was the predominant detected element (32.88 wt%), accompanied by substantially lower levels of Al, Mg, Fe, Sr, P, and other minor elements. SEM further revealed irregular mineral fragments together with a population of smaller particles across the examined fields. These mutually consistent spectroscopic, elemental, and morphological observations provide direct characterization of the actual mineral phase incorporated into the PLA-GM-MS matrices rather than relying solely on the general composition reported for mollusk shells in the literature. Mollusk shell-derived materials have previously been considered mainly as sustainable mineral raw materials, biofillers, or reinforcing components for polymer biocomposites (Azarian and Sutapun, 2022; Gigante et al., 2020; Homavand et al., 2024). In the present system, however, the shell-derived phase additionally functions as a composition-dependent modifier of matrix–medium interaction and gentamicin transport.

FTIR characterization of the hot-pressed PLA-GM and PLA-GM-MS20 matrices provided complementary evidence that the principal chemical features of the constituent phases were retained after processing. Both formulations preserved the characteristic PLA spectral features together with gentamicin-associated contributions, whereas the shell-containing matrix additionally exhibited the expected carbonate-related bands. The absence of new major absorption bands suggests that incorporation of the shell-derived mineral phase primarily resulted in formation of a physically heterogeneous polymer–mineral composite rather than a pronounced chemical transformation of the PLA matrix. This interpretation is consistent with the SEM/EDS evidence of spatially dispersed Ca-rich domains embedded within the continuous polymer phase.

The incorporation of the mineral phase did not alter the overall biphasic character of the release profiles: all compositions retained an initial rapid release stage followed by a slower phase approaching a plateau. Thus, increasing the mineral-phase content changed the extent and rate of gentamicin release without qualitatively altering the overall shape of the release profiles. The observed behavior is compatible with increased aqueous accessibility of the composite, changes in polymer–mineral interfacial pathways, and enhanced accessibility of gentamicin to the release medium. These processes may contribute to the observed release behavior to different extents depending on composite composition and incubation time. This interpretation is consistent with current understanding of hydrophilic compound release from biodegradable polyester carriers, where the initial burst stage, diffusion, water uptake, drug distribution, porosity, and matrix degradation may jointly determine the overall release profile (Chang et al., 2006; Pisani et al., 2019; Yoo and Won, 2020; Fredenberg et al., 2011).

The kinetic modeling provides complementary quantitative information on the composition-dependent release behavior. The simplified Higuchi relationship was applied to the common early-stage interval of 0–4 days, for which the area-normalized release profiles showed good agreement with the square-root-of-time relationship. Comparison with the zero-order model using AICc favored the Higuchi relationship for 13 of the 15 individual specimens, supporting its use as a comparative description of the early release stage. In contrast, when the complete 0–16-day profiles were considered, the asymptotic first-order model provided the best overall fit for all specimens. This difference reflects the progressive curvature of the release profiles as they approach a plateau and demonstrates that a single square-root-of-time relationship is not sufficient to describe the entire release period.

Accordingly, *K*_*H*_ is interpreted in the present study as an apparent Higuchi release constant characterizing the early-stage area-normalized release behavior rather than as an intrinsic diffusion coefficient. This interpretation is consistent with the heterogeneous nature of the hot-pressed PLA-GM-MS matrices and with the broader understanding that release from biodegradable polyester systems may involve several simultaneous processes, including water penetration, drug dissolution and diffusion, pore formation, polymer–drug interactions, and matrix degradation (Higuchi, 1961; Siepmann and Peppas, 2011; Fredenberg et al., 2011).

The progressive increase in *K*_*H*_ with increasing shell powder content was consistent with the corresponding increase in day-16 cumulative release, providing complementary kinetic evidence for composition-dependent modulation of gentamicin transport.

The practical significance of this finding is related to the development of biodegradable materials for the local prevention of implant-associated infections, an area in which implantable antibacterial materials and local therapeutic delivery strategies are receiving increasing attention (Xiang et al., 2025). In the context of modern bioactive polymer composites for bone implants, this is particularly important because the transport-regulating and antibacterial functions of such materials remain less extensively studied than their osteoconductive and mechanical characteristics (Khrustaleva et al., 2026b). The effectiveness of these systems depends on their ability to provide antibiotic release during the critical postoperative period, when the risk of bacterial colonization of the implant surface and biofilm formation is particularly high (Ter Boo et al., 2015; Steadman et al., 2023; Zimmerli and Sendi, 2017). The relevance of this approach is further supported by clinical observations of periprosthetic joint infection after total arthroplasty, where recurrent infection may lead to repeated revision surgeries, the implantation of antibiotic-loaded spacers, and prolonged antibacterial therapy (Konkayev et al., 2024). Because microorganisms within orthopedic biofilms exhibit increased tolerance to antimicrobial therapy and host immune defense mechanisms, the local establishment of therapeutically relevant antibiotic concentrations at the implantation site is of particular importance (Ter Boo et al., 2015; Zimmerli and Sendi, 2017).

At the same time, excessively rapid release may lead to premature depletion of the antibiotic reservoir, whereas insufficiently intensive release may fail to maintain therapeutically relevant concentrations during the most critical early postoperative period. Therefore, the ability to modulate release kinetics without increasing the antibiotic dose is of independent practical interest.

The proposed approach also offers a technological advantage: the release behavior can be adjusted by varying the content of a natural mineral phase without increasing the antibiotic dose, chemically modifying PLA, or substantially complicating material fabrication. This creates opportunities for the development of antibacterial blanks and formable components for bone-oriented medical devices.

The present study was focused on establishing the relationship between mineral-phase content and gentamicin release from compact PLA composite matrices. The involvement of altered matrix–medium interactions and PLA/CaCO_3_ interfacial regions provides a plausible interpretation of the observed behavior; however, elucidation of the underlying physicochemical mechanisms will require dedicated investigation of matrix–medium interactions and structural changes during incubation. The present release experiments were performed at pH 7.4; therefore, the influence of mildly acidic conditions representative of inflammatory or infection-associated microenvironments on gentamicin release and matrix behavior should also be examined in future studies. Such studies represent a logical continuation of the present work and would provide further mechanistic insight into the processes underlying the identified transport behavior.

Overall, the present study demonstrates that gentamicin release from compact hot-pressed PLA composites can be modulated by varying the content of a shell-derived biogenic CaCO_3_-containing phase. The key contribution of this work is the demonstration that shell-derived mineral-phase content can be used to modulate gentamicin transport in compact hot-pressed PLA matrices. In addition, the antibacterial assay confirmed that the hot-pressed gentamicin-containing matrices retained activity against both *S. aureus* and *E. coli* across the investigated range of mineral-phase contents. These findings expand the role of shell-derived mineral material beyond that of a conventional filler and support its use as a functional component for tuning mass transport within biodegradable PLA-based antibacterial composites while preserving antibacterial activity after thermal processing.

## 5 Conclusion

This study demonstrated that gentamicin sulfate release from compact hot-pressed PLA matrices can be modulated by varying the content of a shell-derived biogenic CaCO_3_-containing phase. At a constant antibiotic loading, increasing the fraction of the mineral component was accompanied by a progressive increase in area-normalized release *(Q*_*a*_ *)* and in the apparent Higuchi release constant (*K*_*H*_), indicating a composition-dependent increase in early-stage gentamicin release. Importantly, multivariable analysis showed that mineral-phase content remained significantly and positively associated with mass-normalized gentamicin release after adjustment for specimen thickness, indicating that the observed composition-dependent effect cannot be explained solely by geometric variability among the matrices.

Complementary physicochemical and microstructural characterization provided experimental support for the observed composition–release relationship. FTIR, ICP-AES, and SEM characterization established the shell-derived filler as a Ca-rich carbonate-containing biogenic mineral material, while SEM/EDS analysis of the composite matrices demonstrated spatially dispersed Ca-rich mineral domains within the continuous PLA phase. Increasing shell powder content was also accompanied by progressively greater water uptake, supporting enhanced interaction of the mineral-containing matrices with the aqueous environment. Together, these findings indicate that changes in polymer–mineral organization and aqueous accessibility contribute to the composition-dependent modulation of gentamicin transport.

Antibacterial testing further confirmed that all investigated gentamicin-containing matrices retained activity against *S. aureus* and *E. coli* after hot pressing, demonstrating preservation of antibacterial functionality across the investigated range of mineral-phase contents.

Thus, the shell-derived mineral phase functions not only as a structural filler but also as a functional compositional parameter for controlling mass transport within the PLA matrix. The present study establishes a simple materials-based strategy for tuning both the extent and early-stage rate of gentamicin release without increasing the antibiotic loading or chemically modifying PLA. The combined release, kinetic, water-uptake, and microstructural evidence demonstrates that mineral-phase content can be used to regulate local antibiotic transport in compact biodegradable PLA composites while preserving antibacterial functionality after thermal processing.

## Data Availability

The original contributions presented in the study are included in the article/Supplementary Material, further inquiries can be directed to the corresponding authors.
