# Supplementary material for: Modulating gentamicin release from Poly(lactic acid) composite matrices by tuning shell-derived calcium carbonate content for local antibacterial delivery
Source: Front Bioeng Biotechnol. 2026 Sep 30;14:1976612. doi: 10.3389/fbioe.2026.1976612 (PMC13661975; doi:10.3389/fbioe.2026.1976612)
Supplement: Supplementary file 1 [file DataSheet1.pdf]

(a)

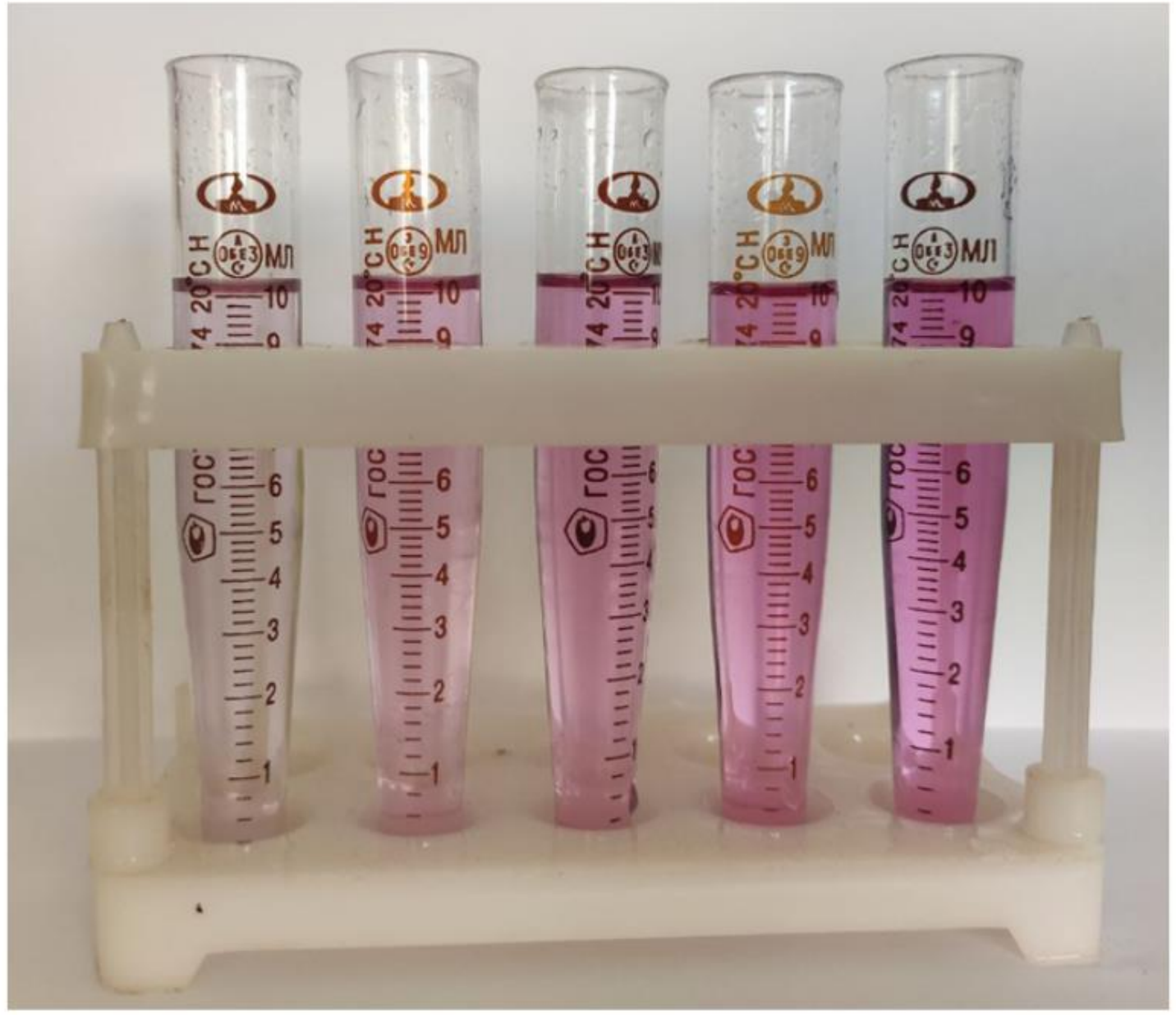

(b)

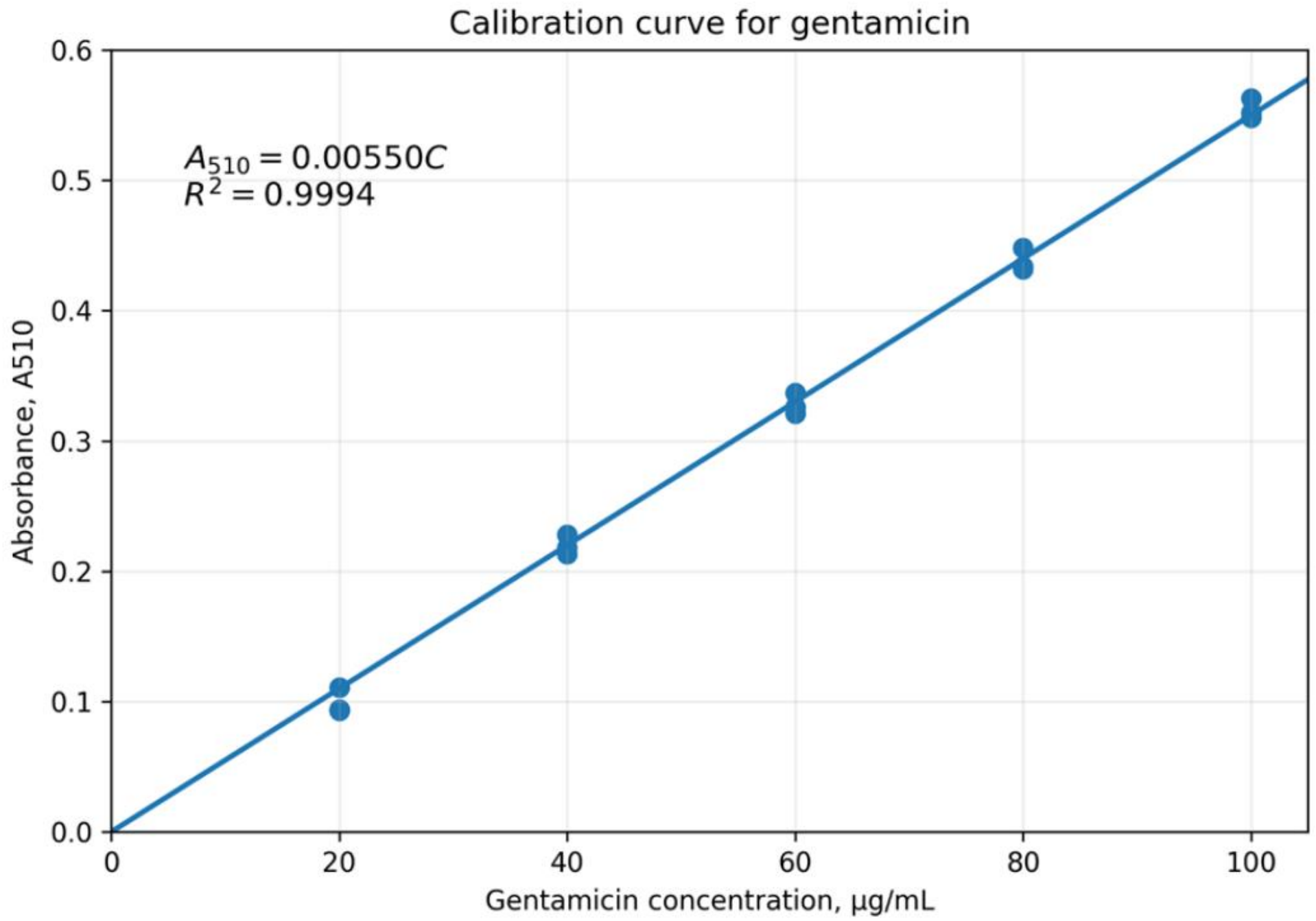

Figure S1. Spectrophotometric calibration of gentamicin sulfate after derivatization with ascorbic acid in DMSO. (a) Visual appearance of gentamicin sulfate standard solutions after derivatization; concentrations from left to right were 20, 40, 60, 80, and 100 µg/mL. The intensity of the pink coloration increased with increasing gentamicin concentration. (b) Calibration curve obtained at 510 nm over the concentration range of 20–100 µg/mL.

Table S1. Calibration data for the spectrophotometric determination of gentamicin sulfate at 510 nm.

Gentamicin concentration, µg/mL	Replicate 1, A510	Replicate 2, A510	Replicate 3, A510	Mean ± SD, A510	RSD, %
20	0.111	0.094	0.093	0.0993 ± 0.0101	10.18
40	0.228	0.213	0.218	0.2197 ± 0.0076	3.48
60	0.326	0.321	0.337	0.3280 ± 0.0082	2.50
80	0.434	0.432	0.448	0.4380 ± 0.0087	1.99
100	0.552	0.548	0.563	0.5543 ± 0.0078	1.40

Supplementary calculation details for gentamicin release

Calculation of cumulative gentamicin release. Gentamicin concentration was calculated from the absorbance measured at 510 nm using the zero-intercept calibration equation $A510 = 0.00550C$.

$$C_{deriv} = A510 / 0.00550$$

$$m_{aliquot} = C_{deriv} \times 5.0 / 1000$$

$$C_{PBS} = m_{aliquot} / 0.1$$

$$M_t = C_t \times V_{PBS} + V_a \times sum(C_i,\ i = 1 \text{ to } n\text{-}1)$$

where C_{deriv} is the gentamicin concentration in the derivatized solution (µg/mL); $m_{aliquot}$ is the gentamicin amount in the analyzed 0.1 mL aliquot (mg); C_{PBS} is the gentamicin concentration in the release medium (mg/mL); M_t is the medium-replacement-corrected cumulative amount released at time t (mg); V_{PBS} is the incubation-medium volume for the individual specimen (mL); V_a is the sampled aliquot volume (0.1 mL); and C_i represents the gentamicin concentration in each previously withdrawn aliquot. Because each withdrawn aliquot was replaced with an equal volume of fresh PBS, V_{PBS} was treated as constant during the cumulative-release calculation.

Normalization used in the main manuscript: $Q_m = M_{16} / m$; $Q_a = (M_{16} / A) \times 1000$, where m is specimen mass (g) and A is the calculated total area of the two major specimen surfaces (mm^2).

Table S2. Specimen-level spectrophotometric data and medium-replacement-corrected cumulative gentamicin release values used to construct the release profiles.

Specimen ID	***Composition***	***Shell powder, wt.%***	***PBS volume, mL***	***Time, d***	***A510***	***C_{deriv}, µg/mL***	***C_{PBS}, mg/mL***	***Gentamicin in medium, mg***	***Previously removed, mg***	***Corrected cumulative M_t, mg***
**1**	PLA-GM	0	8.7	1	0.106	19.2727	0.9636	8.3836	0.0000	8.3836
**1**	PLA-GM	0	8.7	2	0.214	38.9091	1.9455	16.9255	0.0964	17.0218
**1**	PLA-GM	0	8.7	4	0.339	61.6364	3.0818	26.8118	0.2909	27.1027
**1**	PLA-GM	0	8.7	8	0.352	64.0000	3.2000	27.8400	0.5991	28.4391
**1**	PLA-GM	0	8.7	16	0.349	63.4545	3.1727	27.6027	0.9191	28.5218
**2**	PLA-GM	0	9.2	1	0.135	24.5455	1.2273	11.2909	0.0000	11.2909
**2**	PLA-GM	0	9.2	2	0.190	34.5455	1.7273	15.8909	0.1227	16.0136
**2**	PLA-GM	0	9.2	4	0.236	42.9091	2.1455	19.7382	0.2955	20.0336
**2**	PLA-GM	0	9.2	8	0.245	44.5455	2.2273	20.4909	0.5100	21.0009

**2**	PLA-GM	0	9.2	16	0.241	43.8182	2.1909	20.1564	0.7327	20.8891
**3**	PLA-GM	0	9.6	1	0.180	32.7273	1.6364	15.7091	0.0000	15.7091
**3**	PLA-GM	0	9.6	2	0.300	54.5455	2.7273	26.1818	0.1636	26.3455
**3**	PLA-GM	0	9.6	4	0.347	63.0909	3.1545	30.2836	0.4364	30.7200
**3**	PLA-GM	0	9.6	8	0.359	65.2727	3.2636	31.3309	0.7518	32.0827
**3**	PLA-GM	0	9.6	16	0.354	64.3636	3.2182	30.8945	1.0782	31.9727
**4**	PLA-S10-GM	10	9.2	1	0.220	40.0000	2.0000	18.4000	0.0000	18.4000
**4**	PLA-S10-GM	10	9.2	2	0.319	58.0000	2.9000	26.6800	0.2000	26.8800
**4**	PLA-S10-GM	10	9.2	4	0.328	59.6364	2.9818	27.4327	0.4900	27.9227
**4**	PLA-S10-GM	10	9.2	8	0.358	65.0909	3.2545	29.9418	1.0873	31.0291
**4**	PLA-S10-GM	10	9.2	16	0.365	66.3636	3.3182	30.5273	1.4127	31.9400
**5**	PLA-S10-GM	10	10.5	1	0.228	41.4545	2.0727	21.7636	0.0000	21.7636
**5**	PLA-S10-GM	10	10.5	2	0.282	51.2727	2.5636	26.9182	0.2073	27.1255
**5**	PLA-S10-GM	10	10.5	4	0.310	56.3636	2.8182	29.5909	0.4636	30.0545
**5**	PLA-S10-GM	10	10.5	8	0.318	57.8182	2.8909	30.3545	1.0318	31.3864
**5**	PLA-S10-GM	10	10.5	16	0.325	59.0909	2.9545	31.0227	1.3209	32.3436
**6**	PLA-S10-GM	10	9.3	1	0.099	18.0000	0.9000	8.3700	0.0000	8.3700
**6**	PLA-S10-GM	10	9.3	2	0.167	30.3636	1.5182	14.1191	0.0900	14.2091
**6**	PLA-S10-GM	10	9.3	4	0.198	36.0000	1.8000	16.7400	0.2418	16.9818
**6**	PLA-S10-GM	10	9.3	8	0.219	39.8182	1.9909	18.5155	0.6064	19.1218
**6**	PLA-S10-GM	10	9.3	16	0.236	42.9091	2.1455	19.9527	0.8055	20.7582
**7**	PLA-S20-GM	20	10.8	1	0.271	49.2727	2.4636	26.6073	0.0000	26.6073
**7**	PLA-S20-GM	20	10.8	2	0.448	81.4545	4.0727	43.9855	0.2464	44.2318
**7**	PLA-S20-GM	20	10.8	4	0.522	94.9091	4.7455	51.2509	0.6536	51.9045
**7**	PLA-S20-GM	20	10.8	8	0.520	94.5455	4.7273	51.0545	1.6045	52.6591
**7**	PLA-S20-GM	20	10.8	16	0.515	93.6364	4.6818	50.5636	2.0773	52.6409
**8**	PLA-S20-GM	20	10.0	1	0.123	22.3636	1.1182	11.1818	0.0000	11.1818
**8**	PLA-S20-GM	20	10.0	2	0.279	50.7273	2.5364	25.3636	0.1118	25.4755
**8**	PLA-S20-GM	20	10.0	4	0.344	62.5455	3.1273	31.2727	0.3655	31.6382
**8**	PLA-S20-GM	20	10.0	8	0.351	63.8182	3.1909	31.9091	0.9927	32.9018
**8**	PLA-S20-GM	20	10.0	16	0.354	64.3636	3.2182	32.1818	1.3118	33.4936
**9**	PLA-S20-GM	20	9.1	1	0.100	18.1818	0.9091	8.2727	0.0000	8.2727
**9**	PLA-S20-GM	20	9.1	2	0.133	24.1818	1.2091	11.0027	0.0909	11.0936
**9**	PLA-S20-GM	20	9.1	4	0.167	30.3636	1.5182	13.8155	0.2118	14.0273
**9**	PLA-S20-GM	20	9.1	8	0.181	32.9091	1.6455	14.9736	0.5127	15.4864
**9**	PLA-S20-GM	20	9.1	16	0.190	34.5455	1.7273	15.7182	0.6773	16.3955

**10**	PLA-S30-GM	30	10.5	1	0.125	22.7273	1.1364	11.9318	0.0000	11.9318
**10**	PLA-S30-GM	30	10.5	2	0.299	54.3636	2.7182	28.5409	0.1136	28.6545
**10**	PLA-S30-GM	30	10.5	4	0.375	68.1818	3.4091	35.7955	0.3855	36.1809
**10**	PLA-S30-GM	30	10.5	8	0.403	73.2727	3.6636	38.4682	1.0645	39.5327
**10**	PLA-S30-GM	30	10.5	16	0.412	74.9091	3.7455	39.3273	1.4309	40.7582
**11**	PLA-S30-GM	30	11.1	1	0.125	22.7273	1.1364	12.6136	0.0000	12.6136
**11**	PLA-S30-GM	30	11.1	2	0.266	48.3636	2.4182	26.8418	0.1136	26.9555
**11**	PLA-S30-GM	30	11.1	4	0.386	70.1818	3.5091	38.9509	0.3555	39.3064
**11**	PLA-S30-GM	30	11.1	8	0.425	77.2727	3.8636	42.8864	1.0718	43.9582
**11**	PLA-S30-GM	30	11.1	16	0.420	76.3636	3.8182	42.3818	1.4582	43.8400
**12**	PLA-S30-GM	30	8.9	1	0.108	19.6364	0.9818	8.7382	0.0000	8.7382
**12**	PLA-S30-GM	30	8.9	2	0.190	34.5455	1.7273	15.3727	0.0982	15.4709
**12**	PLA-S30-GM	30	8.9	4	0.248	45.0909	2.2545	20.0655	0.2709	20.3364
**12**	PLA-S30-GM	30	8.9	8	0.290	52.7273	2.6364	23.4636	0.7255	24.1891
**12**	PLA-S30-GM	30	8.9	16	0.290	52.7273	2.6364	23.4636	0.9891	24.4527
**13**	PLA-S40-GM	40	7.9	1	0.275	50.0000	2.5000	19.7500	0.0000	19.7500
**13**	PLA-S40-GM	40	7.9	2	0.429	78.0000	3.9000	30.8100	0.2500	31.0600
**13**	PLA-S40-GM	40	7.9	4	0.534	97.0909	4.8545	38.3509	0.6400	38.9909
**13**	PLA-S40-GM	40	7.9	8	0.544	98.9091	4.9455	39.0691	1.1255	40.1945
**13**	PLA-S40-GM	40	7.9	16	0.547	99.4545	4.9727	39.2845	1.6200	40.9045
**14**	PLA-S40-GM	40	10.0	1	0.181	32.9091	1.6455	16.4545	0.0000	16.4545
**14**	PLA-S40-GM	40	10.0	2	0.314	57.0909	2.8545	28.5455	0.1645	28.7100
**14**	PLA-S40-GM	40	10.0	4	0.441	80.1818	4.0091	40.0909	0.4500	40.5409
**14**	PLA-S40-GM	40	10.0	8	0.438	79.6364	3.9818	39.8182	0.8509	40.6691
**14**	PLA-S40-GM	40	10.0	16	0.435	79.0909	3.9545	39.5455	1.2491	40.7945
**15**	PLA-S40-GM	40	11.5	1	0.118	21.4545	1.0727	12.3364	0.0000	12.3364
**15**	PLA-S40-GM	40	11.5	2	0.223	40.5455	2.0273	23.3136	0.1073	23.4209
**15**	PLA-S40-GM	40	11.5	4	0.386	70.1818	3.5091	40.3545	0.3100	40.6645
**15**	PLA-S40-GM	40	11.5	8	0.535	97.2727	4.8636	55.9318	0.6609	56.5927
**15**	PLA-S40-GM	40	11.5	16	0.533	96.9091	4.8455	55.7227	1.1473	56.8700

Table S3. Leave-one-out sensitivity analysis of the multiple linear regression for day-16 mass-normalized gentamicin release (Q_m).

Excluded specimen	Composition	B_W	95% CI for B_W	p for B_W	B_h	95% CI for B_h	p for B_h	R^2	Adjusted R^2	Overall model p

**1**	PLA-GM	0.706	0.442–0.971	0.000106	-44.976	-67.396–-22.556	0.001037	0.775	0.735	0.000271
**2**	PLA-GM	0.644	0.369–0.920	0.000322	-45.231	-68.227–-22.235	0.001196	0.735	0.687	0.000672
**3**	PLA-GM	0.690	0.423–0.957	0.000142	-44.491	-67.752–-21.230	0.001461	0.764	0.721	0.000353
**4**	PLA-S10-GM	0.669	0.409–0.930	0.000146	-46.839	-70.865–-22.813	0.001275	0.761	0.718	0.000381
**5**	PLA-S10-GM	0.664	0.407–0.920	0.000139	-46.890	-69.977–-23.803	0.000946	0.766	0.723	0.000339
**6**	PLA-S10-GM	0.685	0.400–0.969	0.000255	-46.914	-72.679–-21.149	0.002059	0.726	0.676	0.000813
**7**	PLA-S20-GM	0.645	0.405–0.884	0.000099	-41.083	-63.023–-19.144	0.001696	0.768	0.726	0.000325
**8**	PLA-S20-GM	0.674	0.416–0.932	0.000127	-46.116	-69.101–-23.130	0.001036	0.765	0.723	0.000344
**9**	PLA-S20-GM	0.631	0.372–0.889	0.000227	-38.714	-64.207–-13.220	0.006566	0.724	0.674	0.000834
**10**	PLA-S30-GM	0.683	0.417–0.949	0.000148	-46.056	-69.281–-22.831	0.001128	0.759	0.715	0.000400
**11**	PLA-S30-GM	0.669	0.403–0.935	0.000176	-45.537	-68.866–-22.208	0.001264	0.753	0.708	0.000456
**12**	PLA-S30-GM	0.670	0.406–0.934	0.000163	-45.370	-72.263–-18.477	0.003422	0.746	0.700	0.000529
**13**	PLA-S40-GM	0.651	0.351–0.950	0.000567	-44.387	-69.096–-19.679	0.002257	0.693	0.637	0.001511
**14**	PLA-S40-GM	0.771	0.523–1.019	0.000028	-49.979	-70.395–-29.564	0.000221	0.821	0.789	0.000077
**15**	PLA-S40-GM	0.606	0.399–0.813	0.000048	-49.946	-68.140–-31.752	0.000084	0.831	0.800	0.000057

Note. Each model was refitted after exclusion of one specimen (n = 14). Predictors: shell powder content (W, wt.%) and specimen thickness (h, mm). B_W and B_h are unstandardized regression coefficients. Full-model reference: B_W = 0.671 (95% CI 0.422–0.919, p = 0.000075); B_h = −45.539 (95% CI −67.656 to −23.423, p = 0.000744); R^2 = 0.757; adjusted R^2 = 0.716; overall p = 0.00021.

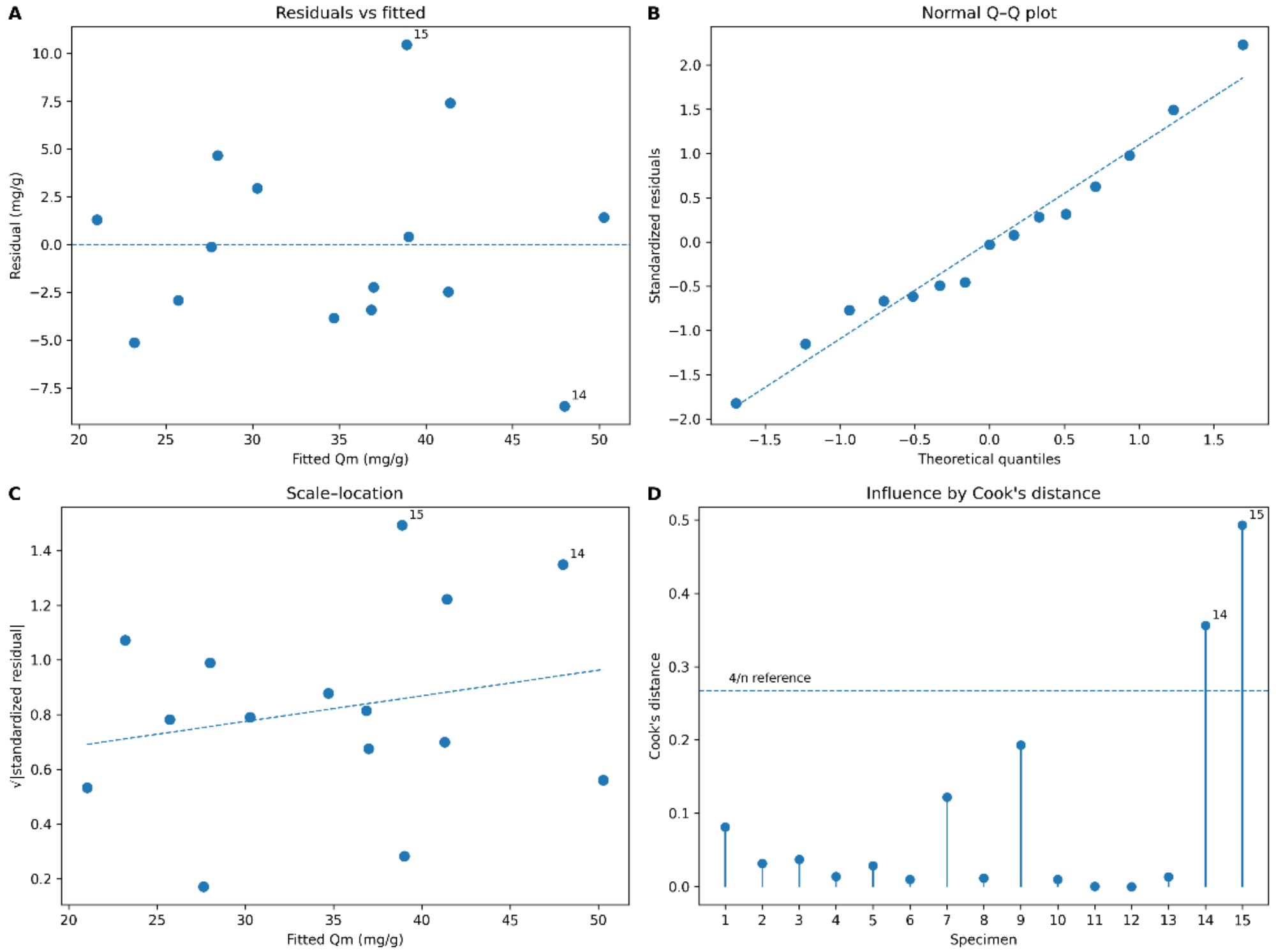

Figure S2. Diagnostic assessment of the multiple linear regression model for day-16 mass-normalized gentamicin release (Q_m). (A) Residuals versus fitted values; (B) normal Q–Q plot of standardized residuals; (C) scale–location plot; and (D) Cook's distance for individual specimens. The dashed line in panel D indicates the 4/n reference value for potentially influential observations.

Table S4. Comparison of the Higuchi and zero-order models for the early-stage gentamicin release profiles (0–4 days)

Sample No.	Composition	K_h, $\mu g \cdot mm^{-2} \cdot h^{-1/2}$	Higuchi R^2	Higuchi RMSE	Higuchi AICc	k_0, $\mu g \cdot mm^{-2} \cdot h^{-1}$	Zero-order R^2	Zero-order RMSE	Zero-order AICc	Lower AICc
**1**	PLA-GM	0.658	0.9465	0.606	−0.005	0.07790	0.9725	0.435	−2.668	Zero-order
**2**	PLA-GM	0.602	0.9872	0.237	−7.528	0.06834	0.7355	1.076	4.589	Higuchi
**3**	PLA-GM	0.689	0.9737	0.396	−3.402	0.07843	0.7462	1.233	5.673	Higuchi
**4**	PLA-S10-GM	0.793	0.9193	0.771	1.918	0.08841	0.5386	1.843	8.889	Higuchi
**5**	PLA-S10-GM	0.851	0.9143	0.837	2.579	0.09455	0.4969	2.028	9.657	Higuchi
**6**	PLA-S10-GM	0.873	0.9788	0.454	−2.325	0.09960	0.7742	1.481	7.142	Higuchi

**7**	PLA-S20-GM	1.470	0.9756	0.815	2.359	0.16730	0.7487	2.613	11.684	Higuchi
**8**	PLA-S20-GM	1.133	0.9489	0.981	3.846	0.13164	0.8692	1.569	7.604	Higuchi
**9**	PLA-S20-GM	0.942	0.9840	0.410	−3.123	0.10669	0.7132	1.740	8.433	Higuchi
**10**	PLA-S30-GM	1.473	0.9413	1.386	6.609	0.17181	0.8843	1.946	9.325	Higuchi
**11**	PLA-S30-GM	1.572	0.9536	1.328	6.269	0.18506	0.9467	1.424	6.828	Higuchi
**12**	PLA-S30-GM	1.535	0.9861	0.664	0.724	0.17735	0.8637	2.082	9.867	Higuchi
**13**	PLA-S40-GM	2.194	0.9902	0.769	1.902	0.25061	0.7887	3.577	14.197	Higuchi
**14**	PLA-S40-GM	1.805	0.9861	0.794	2.150	0.20983	0.9052	2.072	9.827	Higuchi
**15**	PLA-S40-GM	2.024	0.9353	2.082	9.867	0.24092	0.9878	0.905	3.200	Zero-order

Note: The Higuchi and zero-order models were fitted to the same common 0–4-day interval. Both linear models were constrained through the origin. RMSE is expressed in µg/mm². AICc denotes the corrected Akaike information criterion; lower AICc indicates the preferred model among those compared. The Higuchi model showed the lower AICc for 13 of 15 specimens.

Table S5. Comparison of empirical models fitted to the complete 0–16-day gentamicin release profiles

Sample No.	Composition	Higuchi R^2	Higuchi RMSE	Higuchi AICc	Zero-order R^2	Zero-order RMSE	Zero-order AICc	First-order Q_∞, µg/mm²	First-order k_1, h⁻¹	First-order R^2	First-order RMSE	First-order AICc	Best model
**1**	PLA-GM	0.7688	1.370	6.782	0.0638	2.758	15.174	7.678	0.01851	0.9825	0.378	−3.687	First-order
**2**	PLA-GM	0.5704	1.368	6.759	−0.5329	2.584	14.392	5.860	0.03117	0.9995	0.045	−29.234	First-order
**3**	PLA-GM	0.5609	1.597	8.615	−0.5156	2.966	16.048	6.670	0.03112	0.9958	0.157	−14.219	First-order
**4**	PLA-S10-GM	0.5119	1.872	10.523	−0.6573	3.449	17.858	7.741	0.03512	0.9863	0.314	−5.899	First-order
**5**	PLA-S10-GM	0.4006	2.104	11.925	−0.8822	3.728	18.790	7.854	0.04255	0.9918	0.245	−8.854	First-order
**6**	PLA-S10-GM	0.7626	1.665	9.121	−0.1036	3.591	18.341	9.951	0.02123	0.9879	0.376	−3.732	First-order
**7**	PLA-S20-GM	0.5274	3.467	17.920	−0.5766	6.333	25.149	13.942	0.03246	0.9950	0.358	−4.316	First-order
**8**	PLA-S20-GM	0.6814	2.505	14.019	−0.1697	4.800	21.822	11.948	0.02361	0.9786	0.649	2.812	First-order
**9**	PLA-S20-GM	0.6970	1.908	10.750	−0.2926	3.940	19.453	10.184	0.02461	0.9879	0.381	−3.582	First-order

**10**	PLA-S30-GM	0.7518	3.064	16.436	−0.0084	6.175	24.846	16.599	0.02063	0.9811	0.845	5.986	First-order
**11**	PLA-S30-GM	0.7903	3.170	16.846	0.0913	6.599	25.643	18.805	0.01813	0.9894	0.711	3.910	First-order
**12**	PLA-S30-GM	0.8011	2.900	15.775	0.0290	6.407	25.289	18.133	0.01941	0.9984	0.259	−8.226	First-order
**13**	PLA-S40-GM	0.6130	4.856	21.962	−0.4219	9.307	29.769	21.727	0.02884	0.9990	0.252	−8.525	First-order
**14**	PLA-S40-GM	0.6677	3.944	19.467	−0.2393	7.617	27.364	18.732	0.02414	0.9904	0.670	3.194	First-order
**15**	PLA-S40-GM	0.9074	3.587	18.327	0.4864	8.446	28.604	32.806	0.01139	0.9911	1.112	9.276	First-order

Note: Higuchi, zero-order, and first-order models were fitted to the complete 0–16-day release profiles. The first-order model was expressed as $Q_t = Q_\infty \times [1 - \exp(-k_1 \times t)]$. *RMSE is expressed in µg/mm². AICc denotes the corrected Akaike information criterion. The model with the lowest AICc was considered to provide the best empirical description of the complete profile. The first-order model yielded the lowest AICc for all 15 specimens.*

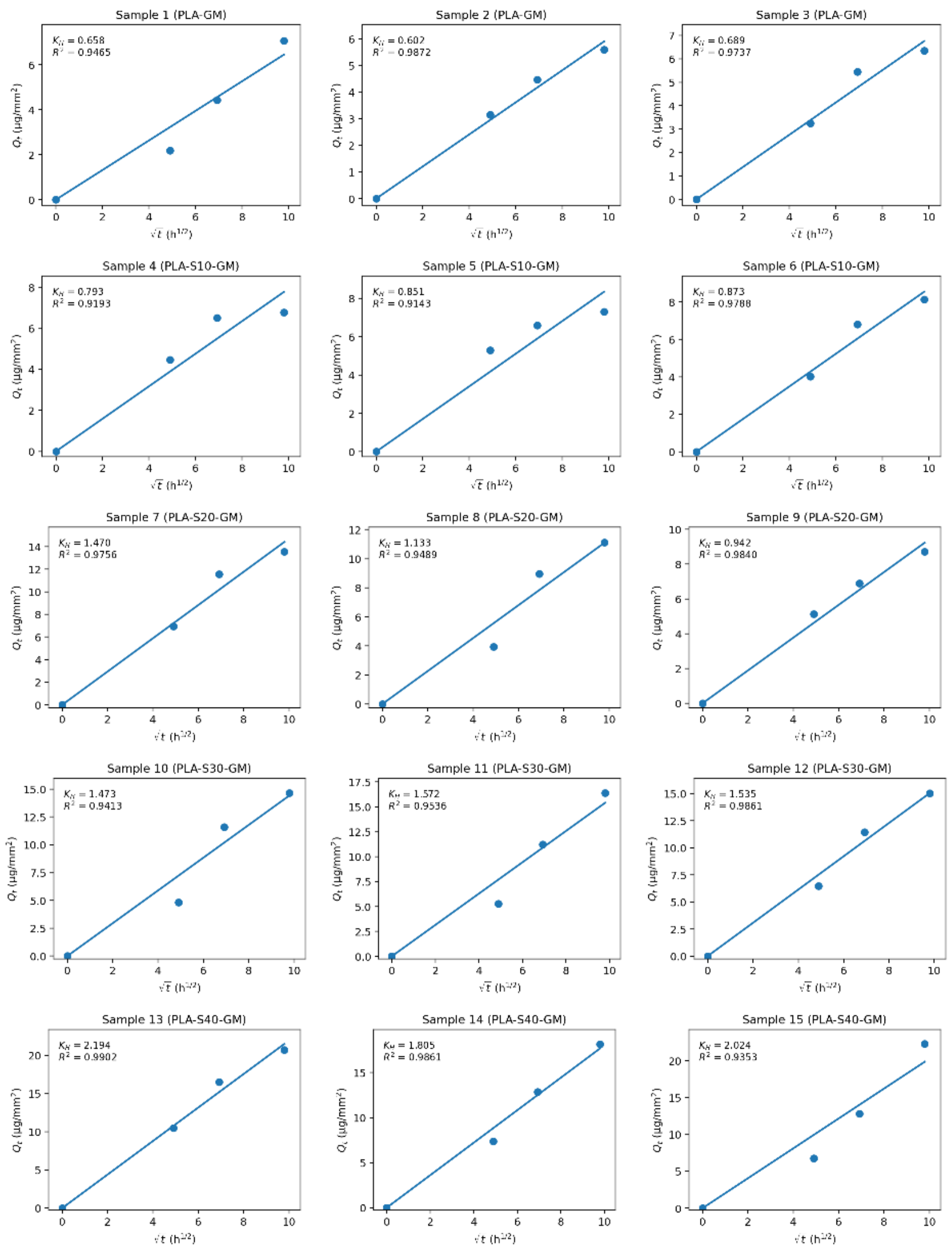

Figure S3. Individual simplified Higuchi fits for the early-stage gentamicin release from PLA/Gen/MS composite matrices. Area-normalized cumulative gentamicin release, Q_t, was plotted against the square root of time over the common 0–4-day fitting interval. Points represent experimental values, and solid lines represent the corresponding Higuchi fits constrained through the origin. The apparent Higuchi release constant, K_h, and coefficient of determination, R^2, are shown for each specimen.

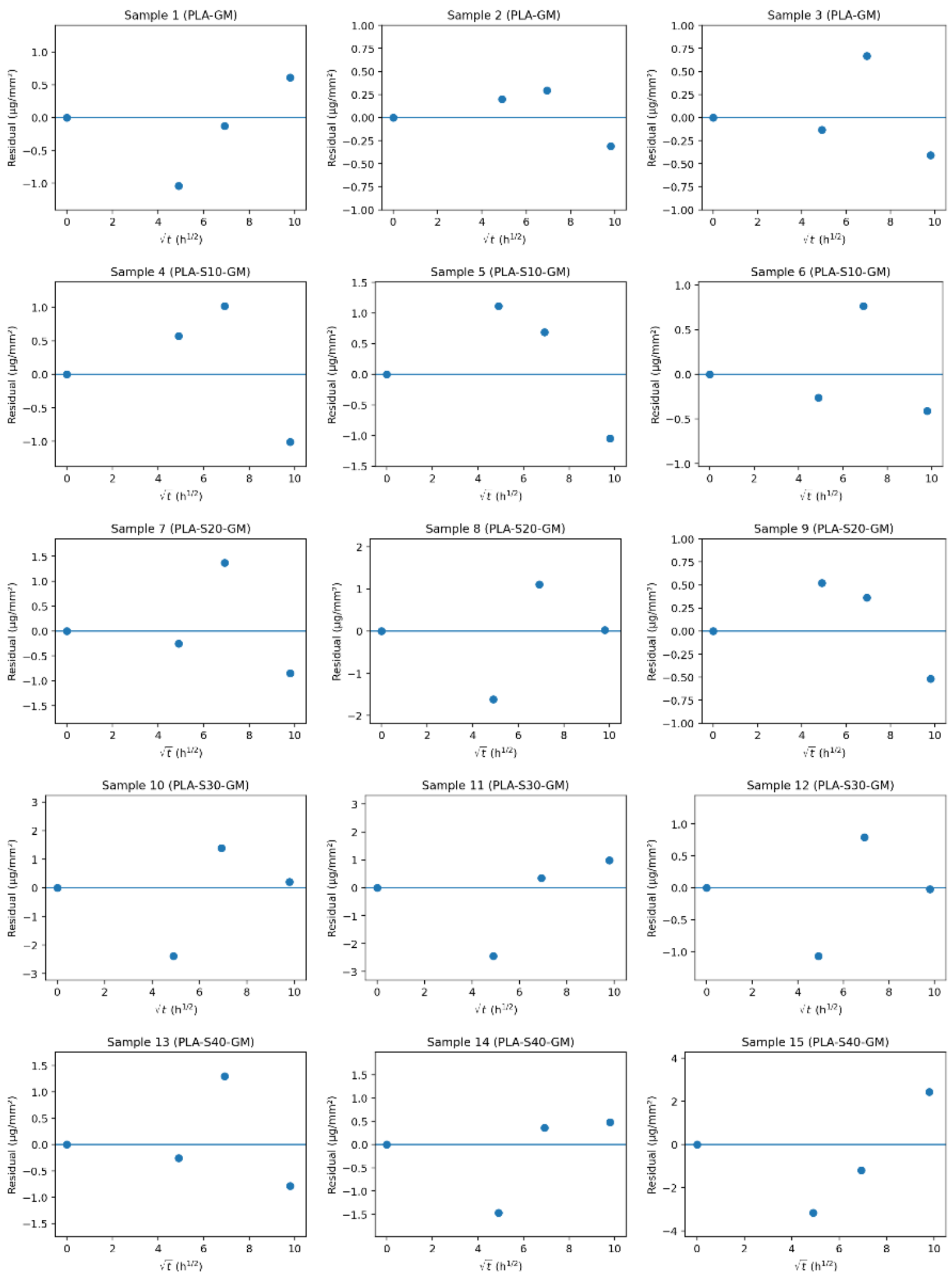

Figure S4. Residual plots for the individual Higuchi fits of early-stage gentamicin release. Residuals were calculated as the difference between experimental and fitted area-normalized release values for the common 0–4-day interval. The horizontal line represents a residual value of zer

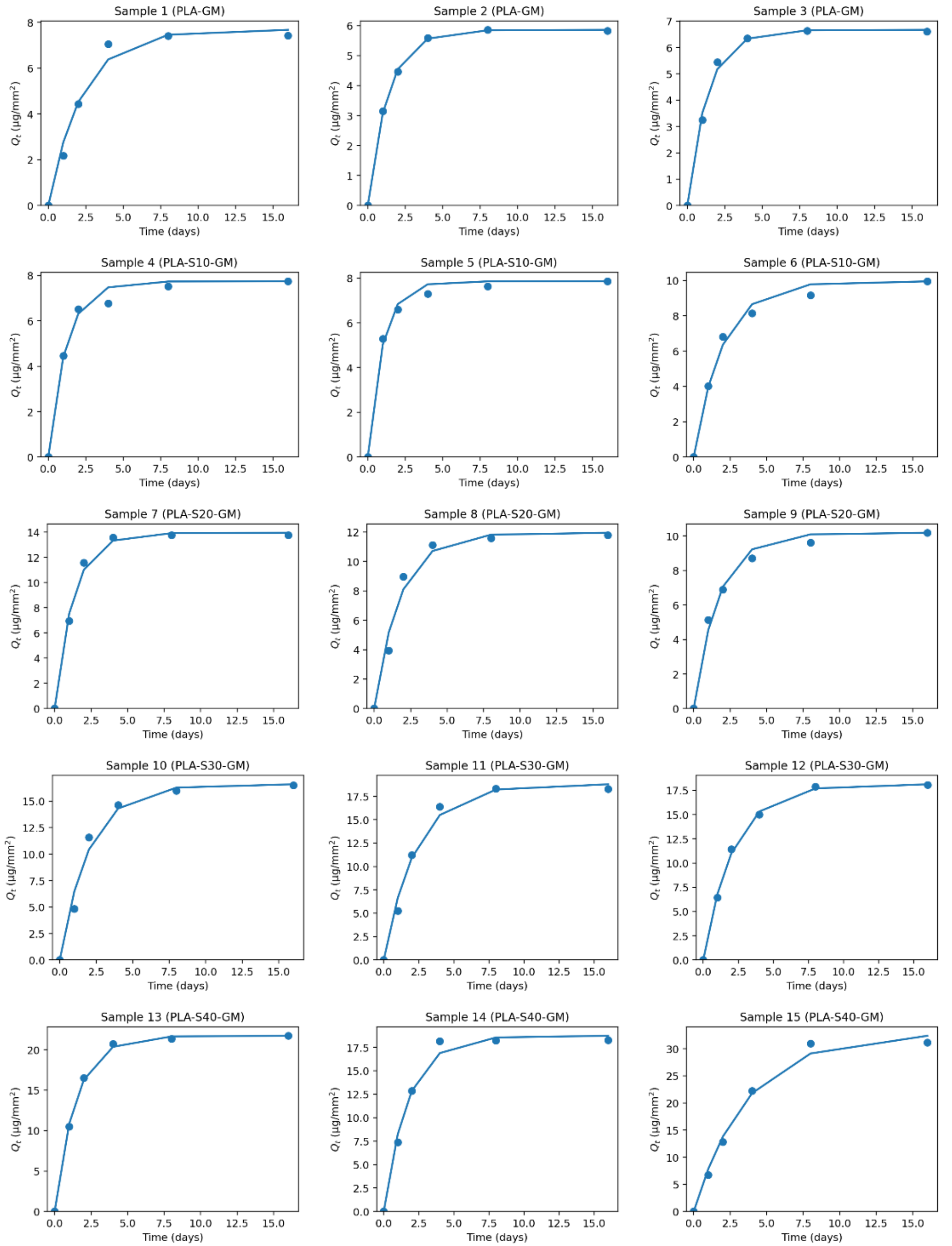

Figure S5. Individual first-order fits for the complete 0–16-day gentamicin release profiles. Points represent experimental area-normalized cumulative release values, Q_t, and solid lines represent the corresponding first-order asymptotic fits over the complete 0–16-day observation period.
